# High-temperature treatments of niobium under high vacuum, dilute air- and nitro­gen-atmospheres as investigated by *in situ* X-ray absorption spectroscopy

**DOI:** 10.1107/S1600577520013557

**Published:** 2021-01-01

**Authors:** Jonas Klaes, Patrick Rothweiler, Benjamin Bornmann, Ralph Wagner, Dirk Lützenkirchen-Hecht

**Affiliations:** a Fakultät 4 – Mathematik und Naturwissenschaften, Bergische Universität Wuppertal, Gauß-Straße 20, 42097 Wuppertal, Germany

**Keywords:** *in situ* EXAFS, high temperature, time-resolved EXAFS, niobium

## Abstract

Niobium metal foils were heat-treated at 900°C under different conditions and *in situ* investigated with time-resolved X-ray absorption fine-structure (EXAFS and XANES) measurements. The present study aims to mimic the conditions usually applied for heat treatments of Nb materials used for superconducting radiofrequency cavities, in order to better understand the evolving processes during vacuum annealing as well as for heat treatments in controlled dilute gases. Annealing in vacuum in a commercially available cell showed a substantial amount of oxidation, so that a designated new cell was designed and realized, allowing treatments under clean high-vacuum conditions as well as under well controllable gas atmospheres. The experiments performed under vacuum demonstrated that the original structure of the Nb foils is preserved, while a detailed evaluation of the X-ray absorption fine-structure data acquired during treatments in dilute air atmospheres (10^−5^ mbar to 10^−3^ mbar) revealed a linear oxidation with the time of the treatment, and an oxidation rate proportional to the oxygen (air) pressure. The structure of the oxide appears to be very similar to that of polycrystalline NbO. The cell also permits controlled exposures to other reactive gases at elevated temperatures; here the Nb foils were exposed to dilute nitro­gen atmospheres after a pre-conditioning of the studied Nb material for one hour under high-vacuum conditions, in order to imitate typical conditions used for nitro­gen doping of cavity materials. Clear structural changes induced by the N_2_ exposure were found; however, no evidence for the formation of niobium nitride could be derived from the EXAFS and XANES experiments. The presented results establish the feasibility to study the structural changes of the Nb materials *in situ* during heat treatments in reactive gases with temporal resolution, which are important to better understand the underlaying mechanisms and the dynamics of phase formation during those heat treatments in more detail.

## Abstract 2

*In situ* X-ray absorption studies of niobium at high temperatures in vacuum, dilute air and nitro­gen atmospheres are reported.

## 1. Introduction   

In many cases, reactive materials need to be processed and *in situ* analyzed at elevated temperatures, *e.g.* during high-temperature brazing of steels (Tillmann *et al.*, 2018 ▸), activation of catalysts (Pu *et al.*, 2017 ▸) or doping of metals and semiconductors (Doering & Nishi, 2008 ▸). In general, special care must be taken in order to prevent unwanted chemical reactions of sensitive samples. Well controlled environments are mandatory to properly process the materials, and to also prevent unwanted reactions during warm-up and cool-down phases. This may be the case if, for example, oxide phases that are thermodynamically unstable at higher temperatures become favourable at lower temperatures, such as, for example, Fe_*x*_O_*y*_ in the case of iron and steels (Ketteler *et al.*, 2001 ▸). In many cases, therefore, (ultra-)high-vacuum (*p* < 10^−6^ mbar) is a mandatory prerequisite for an appropriate thermal processing of many materials.

Here in particular heat treatments of Nb in N_2_ atmospheres for studying high-temperature doping effects are in the focus of interest. Such a nitro­gen doping has been proven to be profitable for the superconducting properties of Nb in accelerating structures (see, for example, Grassellino *et al.*, 2013 ▸; Gonnella & Liepe, 2014 ▸; Gonnella *et al.*, 2015 ▸). Recent experiments have shown that extreme care has to be used for such a high-temperature processing of Nb cavity materials, and that, for example, traces of oxygen, carbon contaminations or remaining hydrogen from outgassing of the vacuum system may substantially alter the near-surface structure and thereby also the superconducting properties of heat-treated Nb materials (Yang *et al.*, 2018 ▸, 2019 ▸; Dhakal *et al.*, 2018 ▸).

Despite the fact that these high-temperature treatments are regularly applied to Nb cavities, there is a substantial lack of information regarding the involved processes on a microscopic level. In particular, a correlation of the structural modifications during the annealing and the resulting superconducting properties is missing. *In situ* experiments under well defined conditions are necessary, and thus X-ray absorption fine-structure spectroscopy (XAFS) was used in the present study. XAFS may provide short-range-order structural information such as coordination distances, coordination numbers and the local disorder, and it also allows the chemical valence of the X-ray absorbing atoms to be determined (see, for example, Koningsberger & Prins, 1988 ▸).

**Figure 1:**
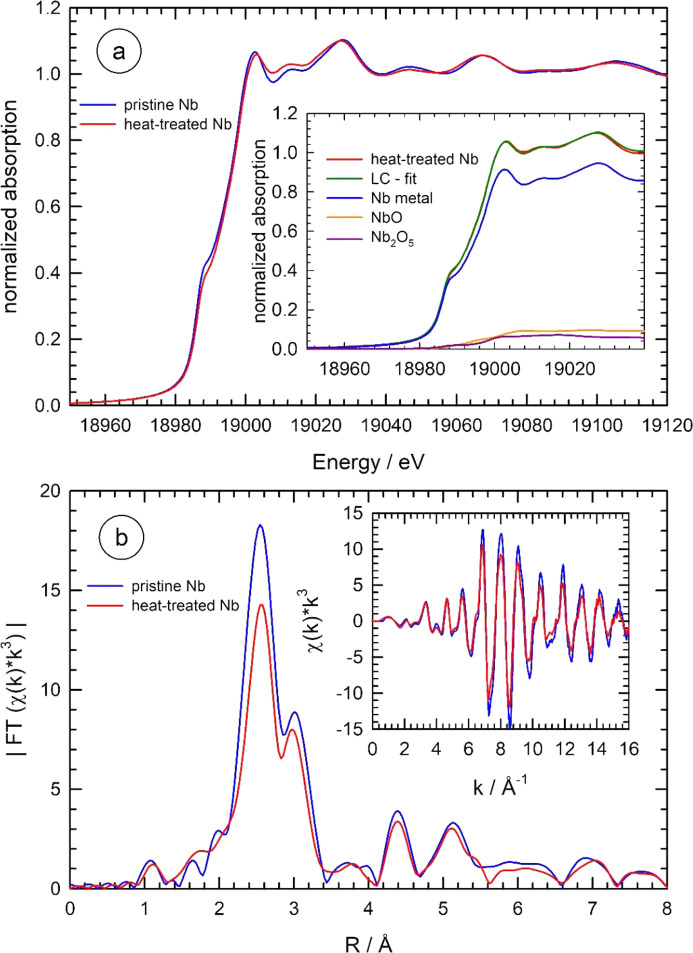
(*a*) XANES spectra of an Nb metal foil (25 µm thickness) at room temperature prior to any heat treatment (blue line) and after a heat treatment in an Anton Paar DHS 1100 cell at 900°C for one hour and cooling-back to room temperature (red line). The inset depicts a linear combination fit of the XANES of the heat-treated sample using reference spectra of an Nb metal foil, NbO and Nb_2_O_5_, leading to 85.0 ± 0.7% Nb metal, 9.3 ± 0.8% NbO and 5.7 ± 0.7% Nb_2_O_5_, respectively. (*b*) Magnitude of the Fourier-transform |FT[χ(*k*)*k* ^3^]| of the *k* ^3^-weighted EXAFS fine structures χ(*k*)*k* ^3^ measured for the pristine Nb foil (blue line) and the heat-treated foil (red line) (*k*-range for the FT: 2.2 Å^−1^ < *k* < 14.5 Å^−1^, data not corrected for phase shifts). In the inset, the *k* ^3^-weighted fine structure oscillations are presented for both data sets.

As a first approach, conventional equipment was used for high-temperature treatments of Nb under vacuum. In particular, the domed hot stage (DHS 1100) from Anton Paar (Kotnik *et al.*, 2006 ▸, see https://www.anton-paar.com/uk-en/products/details/domed-hot-stage-for-four-circle-goniometers-dhs-1100), was employed for our studies. This equipment is regularly available at several synchrotron sources (*e.g.* PETRA III, DESY, Germany, and Photon Factory, KEK, Japan) and has been frequently used for *in situ* X-ray investigations at elevated temperatures (see, for example, Keckes *et al.*, 2001 ▸; Eiper *et al.*, 2004 ▸; Tillmann *et al.*, 2017 ▸; Reiss, 2019 ▸; Wannapaiboon *et al.*, 2019 ▸). However, during treatments of a valve metal like Nb within the DHS 1100, irreversible changes of the Nb samples were already observed when the samples were heated to temperatures of about 400°C under vacuum, *i.e.* without any exposure to additional gases. If the samples are cooled to room temperature after such a treatment for some few minutes only, the measured extended X-ray absorption fine-structure (EXAFS) data revealed a substantial oxidation of the samples. In Fig. 1 ▸, an example of such a heat treatment for one hour at 900°C under vacuum conditions in the Anton Paar DHS 1100 cell is presented. The comparison of X-ray absorption near-edge structure (XANES) data of the pristine Nb foil and the heated foil after cool-down to room temperature reveals small changes both directly visible at the edge as well as in the post-edge region of the XANES [Fig. 1 ▸(*a*)]. A linear combination fit (LCF) using spectra of the Nb metal reference and different Nb oxides suggests the presence of both NbO (Nb^2+^) as well as Nb_2_O_5_ (Nb^5+^) as can be seen in the inset of Fig. 1 ▸(*a*); the spectra of the reference compounds and the determined absorption edge positions are presented in Fig. S1 of the supporting information. The LCF results in about 85.0 ± 0.7% Nb metal, 9.3 ± 0.8% NbO and 5.7 ± 0.7% Nb_2_O_5_, *i.e.* a substantial degree of oxidation is observed even for a 25 µm-thick Nb foil. It should be noted here that the XANES data of the heat-treated sample could also be fitted with a reasonable error using the metal spectrum and only one of the oxides (see Fig. S2 of the supporting information). However, the *R*-factor of the fit substantially improves if NbO is employed (*R* = 1.84 × 10^−4^) instead of Nb_2_O_5_ with *R* = 2.47 × 10^−4^, and the fit quality becomes even superior if both oxides are used (*R* = 1.11 × 10^−4^) [see inset of Fig. 1 ▸(*a*)].

The magnitude of the *k* ^3^-weighted fine structure data |FT[χ(*k*)*k* ^3^]| is presented in Fig. 1 ▸(*b*) for both data sets. Here, the decrease of all detectable coordination shells of the heat-treated sample in comparison with the untreated foil is obvious. In the inset, the χ(*k*)*k* ^3^ data from the two experiments are compared: again the decrease of the amplitudes after the heat treatment is evident, suggesting irreversible changes of the sample, that are well explained by the presence of about 10–15% of Nb oxides according to the XANES LCF analysis. Therefore, meaningful studies of Nb-doping processes at elevated temperatures as mentioned above or other high-temperature treatments of a valve metal like niobium can hardly be performed using the Anton Paar DHS 1100.

In particular for studies of processes employed for thermal treatments of superconducting RF cavities, contaminations such as oxide layers on the surfaces are not tolerable at all, as they substantially degrade the superconducting performance of the devices (see, for example, Malev & Weisser, 1995 ▸; Yang *et al.*, 2018 ▸; Dhakal *et al.*, 2018 ▸; Yang *et al.*, 2019 ▸). All preparation steps and protocols have to be highly reliable, and any oxidation has to be avoided in the processing of cavity materials. Therefore the need for alternative equipment is obvious. In the literature, diverse different set-ups for *in situ* X-ray experiments have been published for different purposes, in gaseous environments as well as in vacuum; reviews have been given, for example, by Chung *et al.* (1992 ▸) and Doronkin *et al.* (2017 ▸). Several cells employ quartz tubes with a thin wall thickness, that can be filled with powder material, and heat is applied from the exterior by hot air, electric heating or infrared light (see, for example, Grunwaldt *et al.*, 2004 ▸; Safonova *et al.*, 2006 ▸; Doran *et al.*, 2017 ▸; Schlicker *et al.*, 2018 ▸). Those set-ups are widely used for *in situ* investigations of catalysts, as the reactive gases can easily be supplied via the quartz tube, and it may be tolerated that the (powdered) samples are in direct contact with the quartz; an overview was recently given by Doronkin *et al.* (2017 ▸). While those cells are easy and comfortable to operate in conjunction with gases or even liquids, major disadvantages arise by the limit of the accessible temperature range, and those cells can hardly be used for massive samples or surface studies using grazing-incidence/grazing-exit X-rays. Furthermore, low pressures may impose problems to the mechanical stability of the capillaries.

Other cell designs have the sample and the X-ray windows well separated from each other, to avoid possible reactions between sample and the window materials, or to protect the windows from excessive heating to keep their temperature sufficiently low. A very elegant approach (see Koziej *et al.*, 2009 ▸; Hübner *et al.*, 2011 ▸) uses integrated microstructures for heating purposes; however, such a design allows only very small sample quantities to be heated. Thus a different layout of the cell is required here.

Murata and co-workers have developed a cell suited for moderate vacuum conditions on the one hand, while employing large X-ray windows for fluorescence mode EXAFS experiments or X-ray diffraction (Murata *et al.*, 2015 ▸). Here, however, the temperature is limited to about 600°C, due to the absence of cooled windows. Other equipment, recently reported by Eigenbrodt *et al.* (2015 ▸), is also suitable for fluorescence EXAFS data acquisition at temperatures of up to 1100°C; however, due to the geometry, this cell appears unsuitable for transmission-mode EXAFS studies, XRD and grazing-incidence X-ray experiments. Cells compatible with our demands mainly use Be windows or domes (*e.g.* Mauron *et al.*, 2011 ▸), a construction material that we want to avoid due to its haza­rdous properties, so that some kind of additional development appears to be required for our purposes, *i.e.* the opportunity to perform transmission-mode spectroscopy and diffraction, as well as apply grazing-incidence X-ray techniques and fluorescence-mode spectroscopy of thin foils and massive bulk samples.

## 2. Cell design   

**Figure 2:**
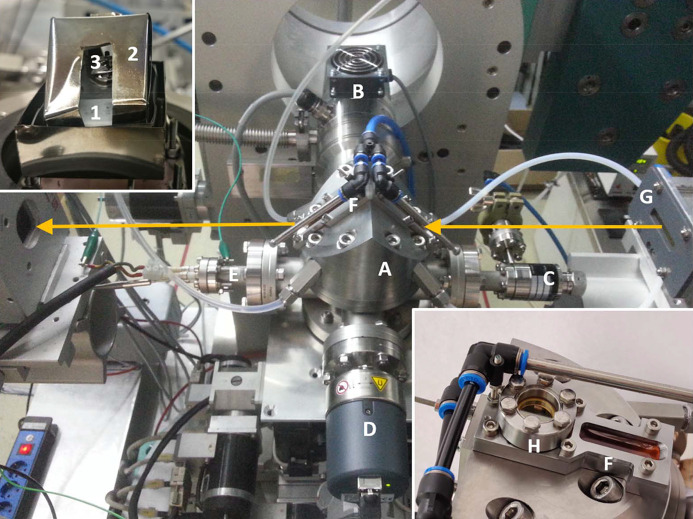
View of the realized cell for X-ray experiments at elevated temperatures mounted on the X-ray diffractometer of DELTA beamline 8. (A) Main body of the cell (dome), with (B) turbomolecular pump, (C) fine leak valve, (D) vacuum gauge, (E) electrical feedthrough for the connection of the heater, (F) air-cooled X-ray windows, (G) ionization chamber for the incident intensity measurements. In the upper inset, the interior of the cell is displayed after the removal of the dome A. The ceramic heating plate (1) with the heat shield (2) and a mounted sample (3) is visible. The X-ray pathway is indicated by orange arrows. In the lower inset, a detailed view of the air-cooled windows is depicted, and in particular the IR-transparent ZnSe-window (H) can be identified.

**Figure 3:**
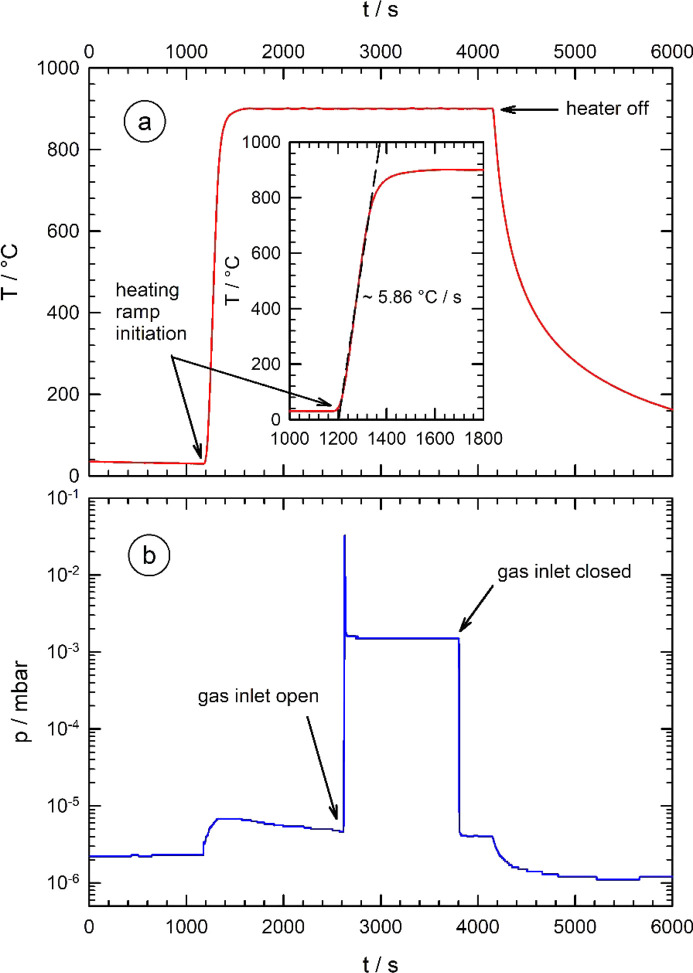
Example of a typical heating process of a niobium metal foil with a well controlled N_2_ gas exposure in the process chamber, comparable with heat treatments of Nb cavities for accelerating structures. (*a*) Evolution of the time–temperature program. At *t* = 1200 s, a heating ramp to *T* = 900°C was initiated, the temperature was then held for ∼3000 s, until the heater was switched off at *t* = 4150 s. In the inset, the fit of the temperature increase yields a heating rate of ∼350°C min^−1^ (dashed black line). (*b*) Evolution of the vacuum conditions during the heat treatment in (*a*). After initiating the heating process, the base pressure of ∼2.2 × 10^−6^ mbar slightly increases during the heat treatment. At *t* = 2600 s, an N_2_ exposure at 1.5 × 10^−3^ mbar was initiated for ∼1200 s. At *t* = 3800 s, the N_2_ exposure was terminated, and the base pressure of the cell (*p* ≃ 1.2 × 10^−6^ mbar) quickly recovered after switching off the heater at *t* = 4150 s.

We have therefore constructed a new cell which is designed for *in situ* X-ray measurements of oxygen-sensitive samples and treatments at elevated temperatures in well defined and controllable reactive and non-reactive gas environments of variable pressure levels from ambient pressure down to a high-vacuum level of 10^−6^ mbar (see Fig. 2 ▸). The cell reveals compact cell dimensions, for use with laboratory equipment and/or easy transportation to synchrotron laboratories and use on small goniometers. It consists of a stainless steel body of about 100 mm outer diameter which houses a ceramic Boralectric heater (Tectra, Frankfurt, Germany) to provide temperatures of up to 1200°C and equipped with two separate molybdenum heat shields for a homogeneous temperature distribution across the samples. For the measurements of thin foils, a molybdenum sample holder was employed (see upper inset of Fig. 2 ▸). Heating rates of more than 300°C min^−1^ are possible, *i.e.* a sample temperature of about 1000°C can be reached within a couple of minutes, while cooling takes much more time. Temperatures of the sample and the heater were measured in parallel by two thermocouples (type K). In Fig. 3 ▸(*a*), a typical example of a heat ramp is shown, similar to the protocols used for high-temperature treatments of Nb cavity materials, with steep heating ramps. Heating is initiated after about 1200 s, leading to a sharp increase of the sample temperature, within 200 s a temperature of about 880°C is reached. In the inset, this steep increase of the temperature is fitted with a linear equation, resulting in an effective heating rate of ∼350°C min^−1^.

Metal-sealed conflat flanges (CF38) are used to connect a small turbomolecular pump (TwisTorr 84 FS, Agilent, Santa Clara, USA) with a membrane backing pump, a full-range penning gauge for vacuum measurements, and electrical feedthroughs for temperature measurements using type-K thermocouples and the electrical supplies of the heater, respectively.

The upper part of the cell, a water-cooled steel dome, is fitted to the body via a CF63 conflat flange with a Viton gasket, so that the insertion of a new sample is easily possible by releasing the related eight screws. The dome is equipped with two large X-ray windows made of Kapton (polyimide), see lower inset of Fig. 2 ▸, with a thickness sufficient for maintaining a base pressure of around 10^−6^ mbar on the one hand while on the other providing a small X-ray attenuation thus enabling experiments down to ∼5 keV photon energy. For an easy exchange, the Kapton windows are sealed against the vacuum vessel with Viton o-rings. For the operation at elevated temperatures, the windows are cooled using pressurized air from the side. Finite-element calculations have shown that thereby the polymer windows do heat up to more that about 50°C, even if the windows are within only 1 cm from the hot parts of the heater and the sample with about 1000°C or more. The induced turbulences of the air are not detectable with X-rays, thus in particular not creating additional noise to the EXAFS data. Typical transmission mode EXAFS data are presented in Fig. S3 of the supporting information, revealing almost noise-free EXAFS oscillations up to more than *k* = 16 Å^−1^.

It is important to note that pressure levels around 10^−6^ mbar can also be guaranteed for sample temperatures of 800°C and more, providing reliable conditions for well controlled sample treatments at elevated temperatures. This can be seen in Fig. 3 ▸(*b*), where the pressure levels monitored during a heat ramp are displayed. While the vacuum conditions prior to the heating are about 2.2 × 10^−6^ mbar, they reach a maximum value of about 6.8 × 10^−6^ mbar at about 1400 s due to the outgassing of the niobium metal foil and the heater itself. However, the pressure slowly decreases to ∼4.5 × 10^−6^ mbar after heating at 900°C for about 1200 s [∼2500 s process time in Fig. 3 ▸(*b*)].

In addition to the X-ray windows, an infrared-transparent ZnSe window of a standard CF16 conflat flange was installed above the X-ray path of the incident beam window, to allow for a temperature control and temperature homogeneity of the sample using an IR camera (FLIR A655sc, FLIR Systems, Inc., Wilsonville, USA), suited for the temperature range from room temperature up to 2000°C with an accuracy of ±2°C. Care must be taken due to the low IR emissivity of niobium at elevated temperatures (Wall *et al.*, 1992 ▸), thus all temperatures reported here were determined using the thermocouples, and the IR camera was only used for the qualitative inspection of the temperature homogeneity here.

A fine leak valve equipped with a pneumatically actuated stop valve serves as well controllable gas inlet, and the heater as well as the gas inlet are remote controlled by a personal computer, so that chemical modifications of the samples induced by the applied gas can be *in situ* observed using X-ray experiments. In Fig. 3 ▸(*b*), the remote-controlled stop valve was opened after about 2600 s process time, and nitro­gen with a pressure of 1.5 × 10^−3^ mbar was admitted for 1200 s. There was a short overshoot of the pressure after the opening of the stop valve for about 7 s, with a maximum pressure of 3.3 × 10^−2^ mbar, leading to a slightly increased gas dose. However, the pressure remains constant within ±0.04 × 10^−3^ mbar during the desired exposure time. Closure of the stop valve results in an immediate decrease of the vacuum level to 4.1 × 10^−6^ mbar within seconds (*t* ≃ 3800 s). Finally, switching off the heater at *t* ≃ 4150 s leads to a full recovery of the vacuum level to values around 1.0 × 10^−6^ mbar after an additional ∼500 s (*t* ≃ 4600 s). It should be stressed again that similar conditions are regularly employed for treatments of superconducting cavities, and therefore the options provided by the presented cell appear useful for systematic investigations of processes such as nitro­gen doping of Nb.

The preparation chamber is lightweight and sufficiently small to fit on a standard X-ray beamline, enabling transmission as well as fluorescence- and reflection-mode EXAFS experiments at elevated temperatures. Samples can be mounted for transmission-mode experiments as well as for grazing-incidence X-ray studies, *i.e.* surface-sensitive reflectometry (Parratt, 1954 ▸) including X-ray fluorescence (Klockenkämper & Bohlen, 2015 ▸) and X-ray standing waves (Zegenhagen, 2018 ▸), EXAFS (Lützenkirchen-Hecht *et al.*, 2016 ▸), asymmetric diffraction (Dangwal Pandey *et al.*, 2018 ▸) as well as small-angle or diffuse X-ray scattering (Kisker *et al.*, 1990 ▸) studies. An example of a combined X-ray reflectometry and grazing-incidence X-ray fluorescence study is presented in Fig. S4 of the supporting information, where a heat treatment of a steel sample under high-vacuum conditions is investigated, and the enrichment of Cr as well as the depletion of Fe in a near-surface region could be successfully monitored.

Furthermore, combined *in situ* transmission mode XANES and X-ray diffraction data measured during the body-centred-cubic to face-centred-cubic phase transition of an iron foil around 915°C are shown in Fig. S5, indicating that combined *in situ* X-ray absorption and diffraction studies at high temperatures using clean high-vacuum conditions are feasible, *e.g.* for investigations of grain growth and grain reorientation, phase transitions and their kinetics during the application of heat.

Here we will focus on *in situ* transmission-mode EXAFS measurements on thin niobium metal foils. A typical processing consists of an annealing treatment in vacuum for 1 h at 900°C, and a subsequent exposure either to high-purity (6.0) N_2_ gas employing pressures in a range from 1 × 10^−3^ to 5 × 10^1^ mbar for varying times, or to oxygen- (air-) atmospheres (10^−5^ to 10^−3^ mbar) to study oxidation dynamics. Finally, the samples were cooled to room temperature either under vacuum or under N_2_ atmosphere. Some selected samples were further cooled down to liquid-nitro­gen temperature (77 K) after their removal from the cell, in order to perform additional XAFS experiments.

## 3. Experimental details   

The experiments presented here have been performed at the SuperXas beamline X10DA (Paul Scherrer Institute, Swiss Light Source, Villigen, Switzerland) (Frahm *et al.*, 2010 ▸) and beamline 8 at the DELTA storage ring (Dortmund, Germany) (Lützenkirchen-Hecht *et al.*, 2009 ▸), both using Si(311) monochromators and Ar gas-filled ionization chambers as detectors, and the Quick-EXAFS endstation P64 at the PETRA III storage ring (DESY, Hamburg, Germany) employing a Si(111) channel-cut monochromator (Bornmann *et al.*, 2019 ▸). EXAFS data at the Nb *K*-edge (18986 eV) were collected prior to any heat treatment as well as during the different process steps at elevated temperatures, and after cooling to room temperature under vacuum. Step-scanning EXAFS measurements taking typically 15–20 min were used for those static samples. The obtained data quality for typical experiments is shown in Fig. S3 of the supporting information.

Time-resolved EXAFS data were *in situ* measured at elevated temperatures within sub-seconds using the Quick-scanning EXAFS opportunities making use of specialized equipment at X10DA (Frahm *et al.*, 2010 ▸) and P64 (Bornmann *et al.*, 2019 ▸). Those techniques are capable of investigating the temporal changes upon heating and the influence of different gas exposures.

Nb metal foils (99.9% purity) of different thicknesses (6 µm, 8 µm and 25 µm) were provided by Goodfellow (Hamburg, Germany), and it is important to note that pristine, untreated niobium metal foils were used for each experiment. A series of samples was investigated after and during heat-treatments in vacuum. High-quality N_2_ gas (6.0) was used for nitro­gen exposures at different pressures adjusted with the fine leak valve, while small pressures of ambient air were admitted to the chamber for oxidation studies. A suitable amount of crystalline Nb_2_O_5_ powder [monoclinic structure (Gatehouse & Wadsley, 1964 ▸), 99.99% purity, Sigma Aldrich] was diluted in boron nitride, pressed to a pellet and measured at the Nb *K*-edge for comparison. Furthermore, niobium nitride material (hexagonal NbN from Alfa Aesar), NbO and NbO_2_ were also investigated, the reference spectra of which are provided in Fig. S1 of the supporting information. X-ray absorption data were analyzed by using the *Athena*/*Artemis*/*Demeter* EXAFS software package (Ravel & Newville, 2005 ▸). In general, the first inflection point of the spectra was used as a measure for the edge position, *E*_0_, and spline functions were used for the background calculation using the algorithms implemented in the *Athena* software, minimizing contributions in the Fourier transform (FT) up to 1.0 (Rbkg = 1.0) if not stated differently in the manuscript. Normalization was performed in the spectral range from 150 eV to 750 eV above the Nb *K*-edge, and the *k*-range from 2.2 Å^−1^ to 14.5 Å^−1^ was subjected to a FT, employing Hanning window functions. For some experiments, the data corresponding to the first two coordination shells (radius *R* in the range 1.5 Å < *R* < 3.4 Å) was back-transformed into *k*-space and fitted with phases and amplitude functions calculated using *FEFF8.4* (Ankudinov *et al.*, 1998 ▸, 2003 ▸), using the body-centred-cubic structure of bulk niobium (space group No. 229, lattice constant of 3.30 Å) as input for the computations. For all the experiments, a niobium metal reference foil was simultaneously measured between the second and a third ionization chamber for a proper energy calibration of the spectra.

## 4. Results and discussion   

### 4.1. Heat treatments in vacuum   

**Figure 4:**
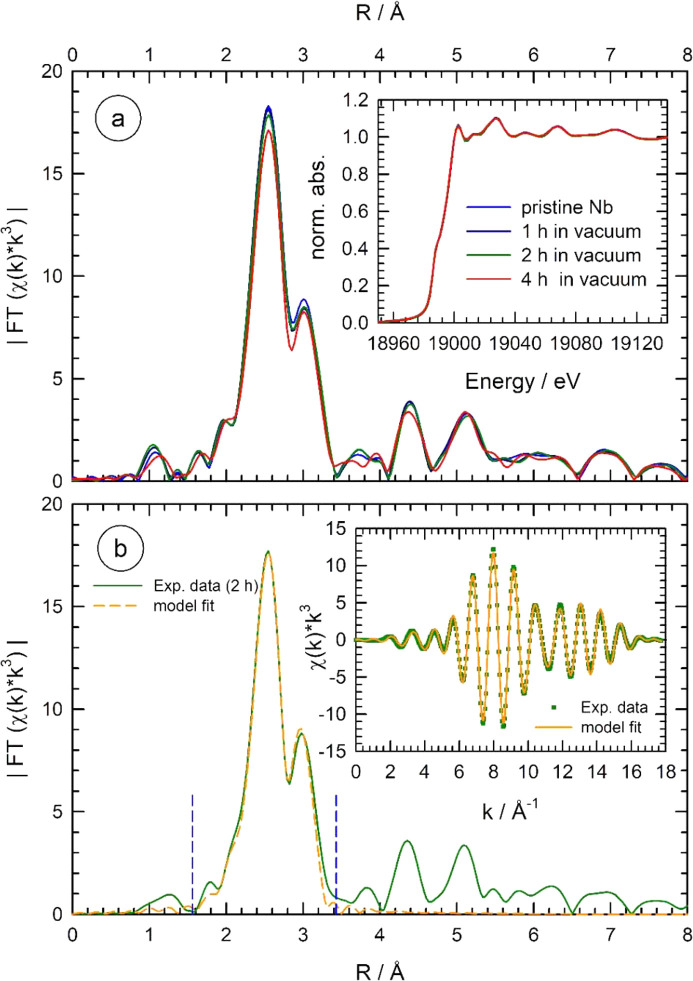
(*a*) Magnitude of the Fourier transform |FT[χ(*k*)*k* ^3^]| of the *k* ^3^-weighted EXAFS fine structures χ(*k*)*k* ^3^ measured for an Nb metal foil (8 µm thickness) at room temperature prior to any heat treatment (blue line) and after a heat-treatment in the new high temperature cell under vacuum conditions close to 10^−6^ mbar for 1 h (dark blue line), 2 h (green line) and 4 h (red line), respectively (*k*-range for the FT: 2.2 Å^−1^ < *k* < 14.5 Å^−1^, the shown data are not corrected for phase shifts). Only very small changes are detectable after 4 h of the heat treatment. In the inset, the related near-edge X-ray absorption spectra are compared with negligible changes after the heat treatments. (*b*) Exemplary fit of the EXAFS data with a two-shell model using the first and second Nb–Nb coordinations of the base-centred-cubic lattice for the sample treated for 2 h under vacuum; the experimental data (green line) as well as the fit (yellow line) are shown. In the inset, the back-transformed *k* ^3^-weighted EXAFS fine structures χ(*k*)*k* ^3^ (data from 1.5 Å to 3.4 Å in the FT as indicated by vertical dotted blue lines) are shown for the experimental data (green squares) and the fit (yellow line). See text for more details.

As a first test of the newly built equipment, Nb foils were treated under vacuum at 900°C for different times. X-ray absorption spectra were recorded prior to the heat treatment, at 900°C as well as after re-cooling to room temperature. In contrast to the heat treatments in the commercial cell, no substantial changes can be derived from the measured EXAFS data, if a heat-treated sample was measured after re-cooling to room temperature. Some exemplary results are shown in Fig. 4 ▸(*a*), where the magnitude of the FT of the χ(*k*)*k* ^3^ data from a pristine Nb sample prior to any heat treatment is compared with spectra obtained after different heat treatments in the range of several hours. As can be seen, after 1 h and 2 h treatments, no distinct changes of the FT are detectable. Only after 4 h of the heat treatment, a slight reduction of the nearest-neighbour coordination peak at 2.6 Å is visible; however, the reduction in amplitude is substantially smaller compared with the 1 h treatment in the commercial cell (compare Fig. 1 ▸). In the inset of Fig. 4 ▸(*a*), the corresponding XANES spectra are shown. In agreement with the EXAFS data, no changes of the pre- and post-edge fine structure and the edge position are detectable, suggesting a negligible degree of oxidation even after prolonged heating at 900°C. Linear-combination XANES fits support this more qualitative observation (see Fig. S6 of the supporting information), *i.e.* for a 1 h treatment in vacuum, no Nb oxide was detectable, while a fraction of only 0.2 ± 0.1% NbO was detectable after 2 h at 900°C, and 1.7 ± 0.6% after 4 h. It should also be noted here that 8 µm-thick Nb foils were used for the heat treatments in the new cell, while a 25 µm foil was used in the commercial setup. Thus, 15% of oxide in the latter case (see Fig. 1 ▸) would correspond to an oxide thickness of more than 3.5 µm, *i.e.* almost 45% of oxidation of a 8 µm foil would have resulted. In contrast, 1.7% NbO as for the 4 h treatment of a 8 µm foil correspond to an oxide thickness of about 0.13 µm only and even less than 20 nm for the 2 h treatment, with an average oxide growth rate of about 35 ± 10 nm h^−1^, allowing quasi oxide-free experimental conditions in the desired time frames of typically 1 h or less. Thus, in conclusion, the new cell clearly offers entirely improved research opportunities.

In order to quantify the structures of the pristine and heat-treated Nb samples in more detail, the first two shells of the body-centred-cubic structure of the Nb lattice (radial distance in the range 1.5 Å < *R* < 3.4 Å in the Fourier transform) were fitted with two individual Nb–Nb coordination shells, modelling the distances (*R*_1_ with eight nearest neighbours, *R*_2_ with six nearest neighbours) and the mean-squared displacements (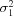, 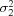). Furthermore, the amplitude reduction factor 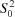 as well as an inner potential shift Δ*E*_0_ were used for the EXAFS fitting, resulting in a total of six independent parameters for each spectrum. A representative fit for a 8 µm-thick Nb sample treated for 2 h in vacuum at 900°C is presented in Fig. 4 ▸(*b*). As can be seen, the fit models the experimental data quite well, both in *R* and in *k*-space [inset of Fig. 4 ▸(*b*)]. From the fit, the following data were determined for the first two Nb–Nb coordination shells of the heat-treat foil: *R*_1_ = 2.850 ± 0.005 Å and *R*_2_ = 3.289 ± 0.007 Å, 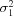 = 6.9 × 10^−3^ ± 0.2 × 10^−3^ Å^2^, and 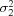 = 7.3 × 10^−3^ ± 0.5 × 10^−3^ Å^2^, and 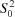 = 0.90 ± 0.08 and Δ*E*_0_ = 3.4 ± 0.8 eV.

**Figure 5:**
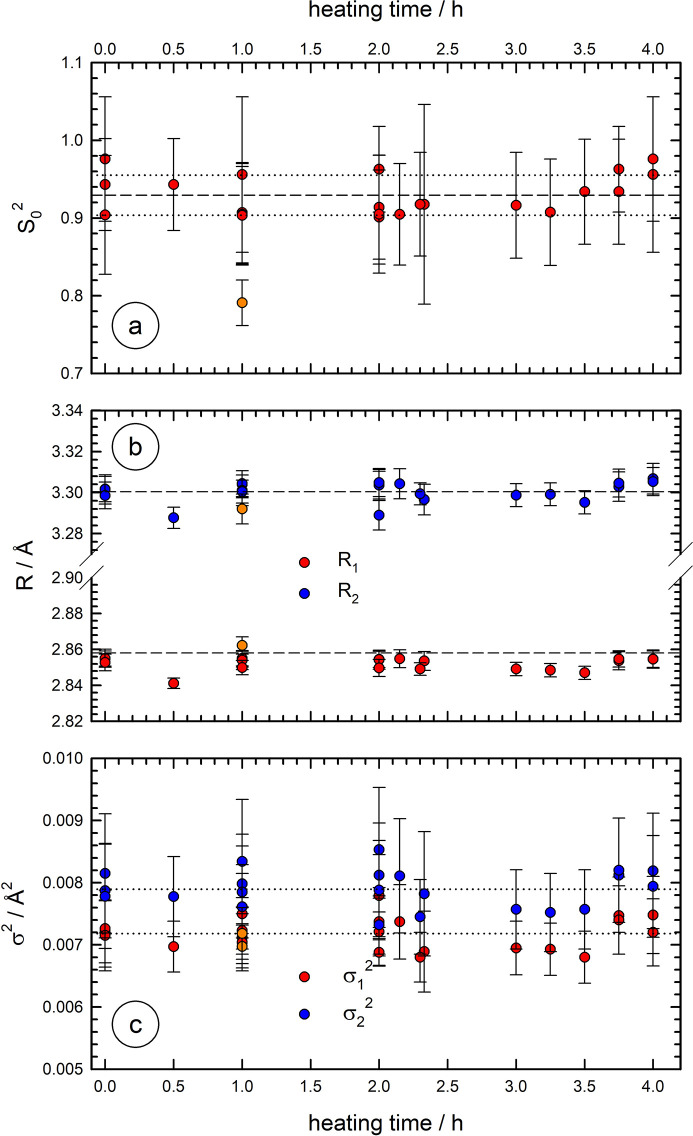
Results of an EXAFS data fitting procedure of Nb foils heat-treated in vacuum as a function of the heating time at 900°C. For the experiments, the samples were cooled to room temperature under high vacuum prior to the measurements. (*a*) Plot of the amplitude reduction factor 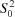; the dashed and the dotted lines represent the average 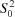 and its confidence interval. (*b*) The distances of the first two Nb–Nb coordination shells (*R*_1_, *R*_2_) in comparison with their literature values (dashed lines) and (*c*) their mean-squared displacements (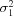, 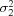) with average values (dotted lines). The orange data points belong to the sample heat-treated in the commercial high-temperature cell. See text for more details.

A systematic evaluation of the Nb samples treated in vacuum at 900°C leads to the data summarized in Fig. 5 ▸; the results obtained from more than 20 individual in-vacuum heat treatments of Nb foils with different thickness are compiled here. As can be seen in Fig. 5 ▸(*a*), the determined values for the amplitude reduction factor 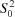 do not systematically vary as a function of the treatment time. If an increasing oxidation of the Nb foils would occur, a systematic decrease of 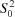 with the time of the heat treatment would be expected. Such a trend is, however, not observed: instead, the 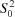 values determined from the fits only show a small scatter of ±0.03 around the average value of 0.93, which further supports the absence of any substantial Nb oxidation during the treatment. For comparison, the X-ray absorption data of the Nb foil heat-treated in the Paar cell (Fig. 1 ▸) was subjected to a similar analysis (see Fig. S7 of the supporting information), leading to a reduced value of 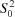 = 0.79 ± 0.03 only. This implies that a neglection of the oxidation would result in erroneous structural information; *i.e.* the analysis of EXAFS data obtained during nitro­gen doping cannot be performed in a straight­forward manner.

Furthermore, also the distances obtained from the fits do not change as a function of the treatment [see Fig. 5 ▸(*b*)], and closely resemble the crystallographic data of bulk Nb, with values of *R*_1_ = 2.858 Å and *R*_2_ = 3.300 Å. The scatter around those values of the order of <0.005 Å is representative for the precision of the EXAFS experiments. As can be seen in Fig. 5 ▸(*c*), the disorder parameters (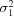, 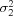) are also almost constant over the entire range investigated, with values of 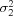 (average 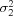 = 7.9 × 10^−3^ ± 0.3 × 10^−3^ Å^2^) slightly larger than those of 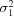 (average 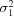 = 7.2 × 10^−3^ ± 0.3 × 10^−3^ Å^2^) for all samples investigated. Assuming an increasing oxidation of the investigated Nb foils with time, also a systematic increase of 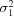 and 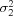 would be expected. These results clearly show that any irreversible alteration of the Nb samples during the annealing in the new cell can be excluded, and that therefore any gas exposure to the samples at elevated temperatures is unambiguously related to the gas supplied. In Sections 4.2 and 4.3, the heated Nb foils were exposed to dilute oxygen (air) and nitro­gen atmospheres.

### 4.2. Heat treatments in air at low pressures   

**Figure 6:**
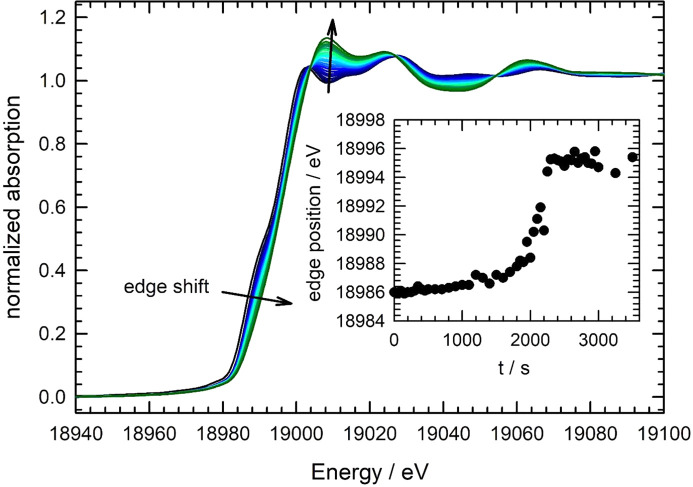
Time-resolved X-ray absorption near-edge spectra (XANES) measured at P64 during the exposure of an Nb thin foil (8 µm thickness) to an air-atmosphere of 10^−3^ mbar at 900°C. The displayed spectra were recorded with 0.5 s per scan (1 Hz), and one spectrum is displayed every three minutes (colour code from dark blue to dark green with increasing time). In the inset, the shift of the absorption edge defined as the first inflection point of the spectrum is shown.

In Fig. 6 ▸, X-ray absorption near-edge spectra (XANES) obtained *in situ* during the exposure of a heated Nb foil (900°C) to 10^−3^ mbar air are displayed. The stop valve was opened after a pre-heating phase of 1 h under vacuum (see Section 4.1), and the changes of the spectra with air exposure time markedly indicate the oxidation of the Nb foil by the application of a small oxygen partial pressure. This can clearly be seen by the shift of the absorption edge to larger energies by about 10 eV (see inset of Fig. 6 ▸), and the increase of the white line intensity at about 19008 eV with the time of the air exposure.

**Figure 7:**
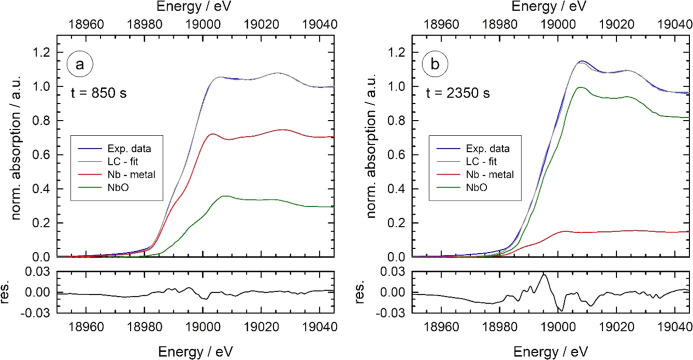
Examples of linear-combination fits using Nb metal and NbO for the modelling of the experimental XANES data. (*a*) *t* = 850 s, yielding 30.7 ± 0.3% NbO and 69.3 ± 0.3% of Nb metal. (*b*) *t* = 2350 s, with 85.6 ± 0.7% NbO and 14.4 ± 0.7% of Nb metal. In the lower panels, the difference between the experimental data and the fits are presented.

For a quantitative analysis of the spectra, it is important to note that isosbestic points are existing for various energies, *e.g.* 19004 eV, 19027 eV, 19054.5 eV and 19098 eV, indicating that only two different niobium species are contributing to the measured data. This finding is supported by a principal component analysis (Malinowski & Howery, 1980 ▸; Ressler *et al.*, 2000 ▸), which yields that only two independent components (*i.e.* Nb species) are needed to describe all the measured XANES data by linear combination fits. According to the experiments presented above (Section 4.1), it is clear that the initial spectra belong to the pure Nb metal species at 900°C. Thus the remaining task is the identification of the second Nb species and a reconstruction of the measured data by means of linear combination fits. We have therefore tested near-edge spectra of Nb_2_O_5_ (Nb^5+^), NbO_2_ (Nb^4+^) and NbO (Nb^2+^) for the modelling of the data. The XANES spectra of those Nb oxides show substantial differences at the edge and in the post-edge region (see, for example, Sakamoto *et al.*, 2015 ▸), thus sensitively allowing their discrimination. It appears that by using the XANES of NbO, the fit errors and the uncertainties for the compositions are substantially smaller compared with the other oxides. Two representative fits for oxidation times of 850 s and 2350 s, *i.e.* in the initial phase and the final phase of the experiment, are shown in Fig. 7 ▸, revealing the high quality of the fits. For comparison, exemplary fit results obtained with NbO_2_ and Nb_2_O_5_ are presented in Fig. S8 of the supporting information. As can be seen there, an overall worse fit quality results, with substantially larger deviations between the experimental data and the best possible fits. Numerical values for the resulting fit residuals are *R* = 3.5 × 10^−3^ for the fit using Nb metal and Nb_2_O_5_, *R* = 2.6 × 10^−3^ for the fit using Nb metal and NbO_2_, and *R* = 4.0 × 10^−4^ for the fit using Nb metal and NbO, respectively.

An Nb^2+^ oxidation state appears to be in contradiction with the thermodynamic data of Nb oxides (see Jacob *et al.*, 2010 ▸), which would favour the formation of Nb^5+^ at elevated temperatures instead (*i.e.* Nb_2_O_5_). However, the results are well in agreement with X-ray diffraction results, showing that NbO is a major constituent of the oxide scale during the oxidation in air (Clenny & Rosa, 1980*a* ▸; Bouillet *et al.*, 1997 ▸); however, those experiments were performed at substantially larger pressures in the range from 10^−2^ mbar to 1000 mbar, and NbO_2_ and Nb_2_O_5_ were always detected in parallel to NbO in those experiments. Furthermore, it is reported in those works that NbO is predominantly formed during the first few minutes of the oxidation, and the amounts of NbO_2_ and Nb_2_O_5_ phases decrease with decreasing oxygen pressure. For temperatures between 300°C and ∼700°C, a dry oxidation (20 mbar O_2_) of Nb(110) single crystals leads to the formation of epitaxial NbO layers at the surface (Hellwig & Zabel, 2000 ▸; Delheusy, 2008 ▸). Thus, the formation of NbO_2_ and Nb_2_O_5_ appears to be further suppressed by applying substantially lower air (oxygen) pressures and an increased temperature in the present study. The formation of a pure NbO oxide on top of the Nb metal was also confirmed by photoemission (X-ray photoelectron spectroscopy and ultraviolet photoelectron spectroscopy) studies; however, only during the initial stages of oxidation with very thin oxide layers of some few nanometres only (Lindau & Spicer, 1974 ▸; Hu *et al.*, 1989 ▸).

**Figure 8:**
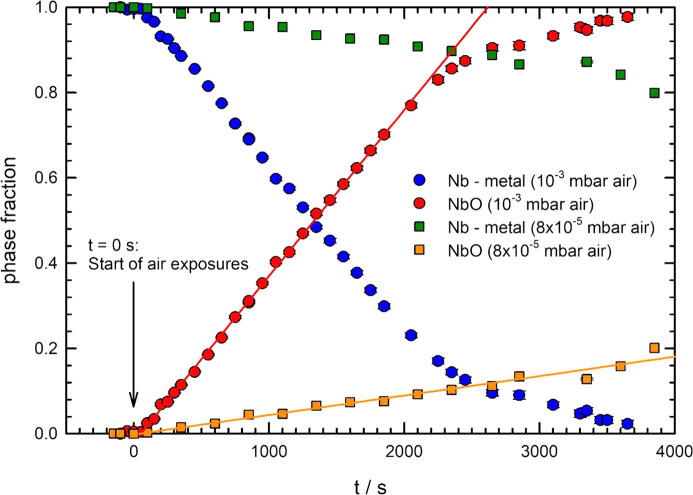
Concentration of Nb-metal (blue circles, green squares) and NbO (red circles, brown squares) as determined from the evaluation of time-resolved EXAFS data of 8 µm-thick Nb foils as a function of time and two different air pressures during oxidation at 900°C. The full coloured lines correspond to linear oxidation kinetics with time for both treatments.

Similar X-ray absorption experiments were also performed for different air pressures during the oxidation in the range from 10^−5^ to 10^−3^ mbar, and the results are compiled in Fig. 8 ▸, where the concentration of Nb metal and NbO are plotted as a function of the oxidation time. As can be seen, oxidation at 10^−3^ mbar air leads to an almost linear increase of the NbO concentration, in good agreement with previous results (Bridges & Fassell, 1956 ▸; Kofstad & Kjollesdal, 1961 ▸; Mukaibo *et al.*, 1965 ▸; Clenny & Rosa, 1980*a* ▸). The oxidation rate substantially increases with the air pressure as can be seen by the variation of the slopes in Fig. 8 ▸ for the two pressures of 10^−3^ mbar and 8 × 10^−5^ mbar, reflecting the strong influence of the larger oxygen exposure on the oxide growth rate. From the thickness of the used foil, oxidation rates of ∼(0.187 ± 0.001) µm min^−1^ and (0.022 ± 0.001) µm min^−1^ were estimated for 10^−3^ mbar and 8 × 10^−5^ mbar air, respectively. Additional experiments at even lower oxygen partial pressures around 1 × 10^−5^ mbar indicated that a slight oxidation (around 1–2%) occurs on time scales of 30 min, corresponding to an oxide growth rate of some few nanometres per minute, *i.e.* there is clear evidence that even extremely low oxygen pressures lead to a substantial oxidation of Nb at temperatures of 900°C. On the other hand, as the vacuum pressure within the newly developed cell is around 2 × 10^−6^ to 3 × 10^−6^ mbar even for temperatures in the range 800–900°C, such an oxide growth can be further suppressed to uncritical values, as discussed in Section 4.1. These results are of special importance for all treatments of niobium at elevated temperatures, in particular for the nitridation of superconducting radiofrequency Nb cavities [N-doping (Grassellino *et al.*, 2013 ▸, Gonnella & Liepe, 2014 ▸; Gonnella *et al.*, 2015 ▸; Yang *et al.*, 2019 ▸)], where the pre-heating treatments of the cavities should be carried out using best-possible vacuum conditions according to the results presented here.

### 4.3. Heat treatments in nitro­gen   

**Figure 9:**
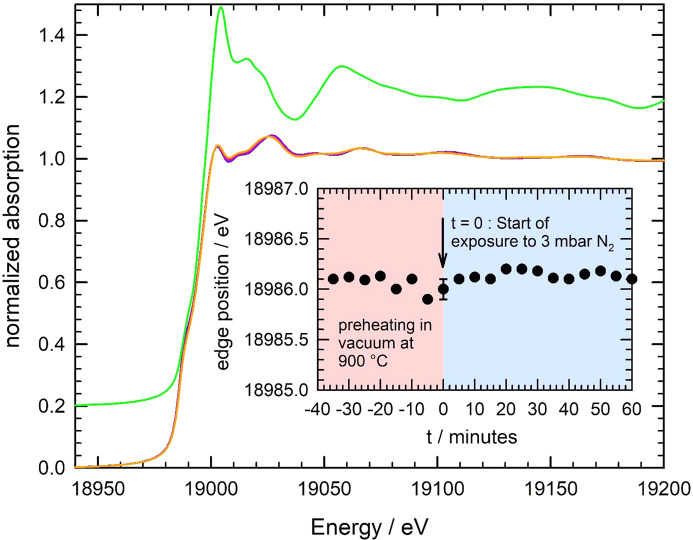
*In situ* detected XANES spectra at the Nb *K*-edge measured during the exposure of a stack of two Nb foils (6 µm thickness each) to a nitro­gen atmosphere of 3 mbar at 900°C. The displayed spectra were recorded with 0.5 s per scan (1 Hz), and one spectrum is displayed every five minutes (colour code from dark blue via violet and red to orange with increasing time). The XANES spectrum of the NbN reference material is shown with an offset of +0.2 for a better comparison (green line). In the inset, the determined absorption edge position (defined as the first inflection point of the spectrum) is shown as a function of the exposure time, *i.e.* *t* = 0 corresponds to the start of the N_2_-exposure.

**Figure 10:**
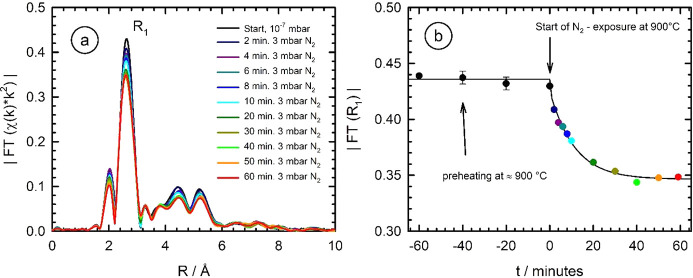
(*a*) *In situ* detected structural changes during the N_2_ exposure (3 mbar) of a stack of Nb foils (2 × 6 µm thickness) at 900°C as a function of time. A clear reduction of all peaks in the magnitude of the Fourier-transformed *k* ^2^-weighted EXAFS data FT|χ(*k*)*k* ^2^| are detectable (*k*-range for the FT: 2.2 Å^−1^ < *k* < 10.8 Å^−1^, the data are not corrected for phase shifts). The intensity of the peak related to the nearest neighbour |FT(*R*_1_)| is plotted as a function of the process time in (*b*). After 60 min preheating under vacuum at 900°C, the sample was exposed to an atmosphere of dried N_2_ gas at 3 mbar. Black circles correspond to the pre-heating phase under high-vacuum conditions, while the coloured data points in (*b*) belong to the treatment in 3 mbar N_2_ according to the legend in (*a*). For *t* > 0, the fit line corresponds to an exponential decay with time.

Finally, some preliminary X-ray absorption spectroscopy investigations have been performed during the nitro­gen exposure of Nb metal foils. In Fig. 9 ▸, XANES spectra measured during the high-temperature treatment of niobium metal in an N_2_ atmosphere of 3 mbar at 900°C are presented. The samples were first pre-heated at 900°C under vacuum conditions for 1 h, and the nitro­gen exposure is subsequently initiated for 60 min at *t* = 0. First of all, no substantial changes of the XANES are visible in the edge region as a function of time, which quantitatively agrees with the determined edge positions, that do not systematically deviate from the edge energy of a Nb reference foil (*E* = 18986.0 ± 0.1 eV, see inset). By comparison with the XANES spectrum of the NbN reference, it is obvious that, for example, a substantial increase of the white line intensity should be expected if NbN is present in significant a mounts. Due to the use of very thin Nb foils here, an inherent sensitivity for the presence of surface modifications is given, and thus it can be anticipated that no substantial amounts of Nb nitride (NbN) with an edge energy of 18994 eV (see Fig. S1) are formed. Furthermore, only very subtle changes of the post-edge absorption fine structure are detectable, that may be explained by the incorporation and dissolution of nitro­gen within the Nb lattice. Accordingly, small changes of the FTs are measured as a function of the nitro­gen exposure time as depicted in Fig. 10 ▸(*a*). Here we have used the *k* ^2^-weighted absorption fine structure, and due to the fast decay of the EXAFS oscillations with increasing temperature (see Fig. S3 of the supporting information), only the *k*-range from 2.2 to 10.8 Å^−1^ was subjected to a FT.

As can be seen in Fig. 10 ▸(*a*), a clear reduction of all Nb–Nb coordination shells occur on time scales of some few minutes, and a saturation occurs after about 50 min of nitro­gen exposure with a pressure of 3 mbar. This can be more clearly seen in Fig. 10 ▸(*b*), where the magnitude of the first Nb–Nb coordination in the Fourier transform |FT(*R*_1_)| is plotted as a function of the heating time in the nitro­gen atmosphere. While |FT(*R*_1_)| is constant in the preheating treatment under vacuum, as can be expected from the results presented in Section 4.1, a distinct, sharp decrease of |FT(*R*_1_)| is detected within the first few minutes of the nitro­gen exposure induced by the remote-controlled opening of the inlet valve (compare Fig. 3 ▸). From the presented results, any niobium nitride formation can be excluded, as this should lead to prominent peaks in the radial distance range of about 1.5–1.6 Å in the FT. Furthermore, a positive shift of the absorption edge is not observed (see Fig. 9 ▸). Similar to the oxidation experiments presented in Fig. 6 ▸, however, such a shift is expected for Nb_*x*_N_*y*_ compounds due to the changed chemical valency of niobium. Therefore, we cannot give evidence for the formation of Nb_*x*_N_*y*_ in substantial amounts from the EXAFS and XANES data presented here.

In any case, the doping seems to reach a saturation after about 35 min of the N_2_ exposure, as the amplitudes of the Nb *K*-edge EXAFS are not decreasing further for larger times. From the decrease of |FT(*R*_1_)| in comparison with the original value prior to the nitro­gen exposure, an average concentration of about 1 at.% N may be calculated from a more detailed modelling of the EXAFS data (Rothweiler *et al.*, 2021 ▸). Such a value is well in accordance with equilibrium values given in the literature (Brauer & Esselborn, 1961 ▸; Fromm & Jehn, 1969 ▸). Furthermore, assuming a diffusion coefficient of the order of *D* ≃ 10^−9^ cm^2^ s^−1^ for nitro­gen in niobium and the temperatures applied here (Powers & Doyle, 1959 ▸; Clenny & Rosa, 1980*b* ▸; Keinonen *et al.*, 1984 ▸), a diffusion length *x* = 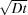 of about 12 µm can be estimated for an exposure time of 30 min. Such a value corresponds well to the thickness of the used Nb foil, *i.e.* the observed saturation of the nitro­gen uptake by the Nb foil [Fig. 10 ▸(*b*)] might be qualitatively explained by the uptake and the diffusion of N-atoms in the Nb foil.

**Figure 11:**
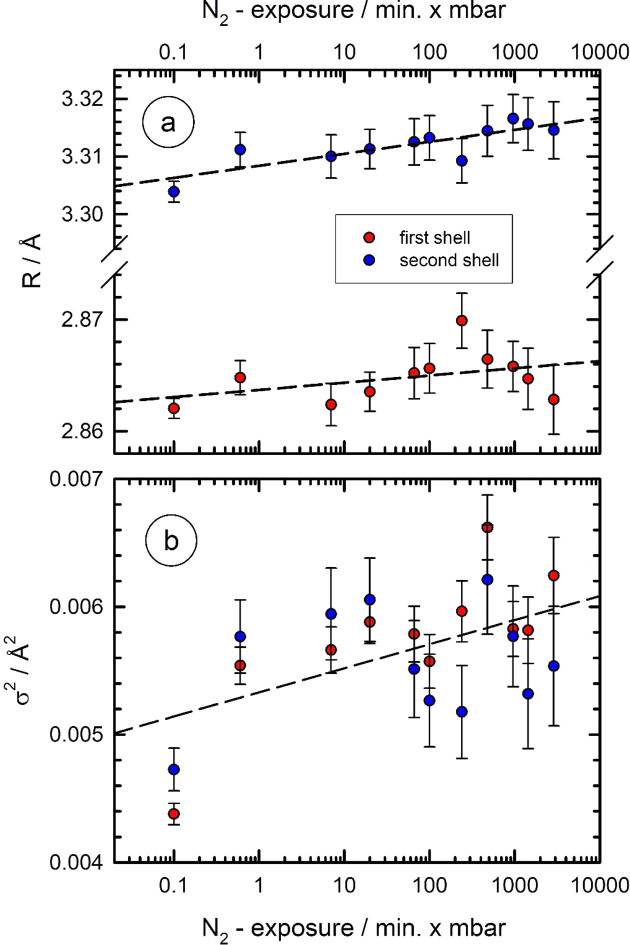
Results of an EXAFS data fitting procedure of Nb foils heat-treated in nitro­gen atmospheres at 900°C for different pressures and times as a function of the N_2_ exposure, *i.e.* the product of the N_2_ pressure and the time of the treatment in N_2_. For the measurements, the samples were cooled to liquid-nitro­gen temperature (∼80 K) to reduce thermal vibrations and to increase the accuracy of the fit results. (*a*) Plot of the distances of the first two Nb–Nb coordination shells (*R*_1_, *R*_2_), dashed trend lines show the increase of the determined bond distances with N_2_ exposure, and (*b*) mean-squared displacements (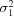, 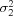) with a common trend line for both shells. See text for more details.

In order to analyse the structural changes of Nb induced by the heat treatment in nitro­gen atmospheres in more detail, we have analysed some of the nitro­gen-treated samples after their removal from the high-temperature cell, and cooling down to liquid-nitro­gen temperature (∼80 K) in a suitable cryostat. Ex situ EXAFS data were subsequently measured for different nitro­gen exposures, measured as the product of the nitro­gen pressure during the treatment and the time of the nitro­gen treatment. We have performed a similar analysis as for the vacuum-annealed samples, *i.e.* again a two-shell fit was performed, the results of which are compiled in Fig. 11 ▸. As can be seen, the Nb—Nb bond distance of the first and second shell slightly increase with the nitro­gen exposure, with values of about 1.6 × 10^−3^ Å per decade of the nitro­gen exposure for the first shell and 2.1 × 10^−3^ Å per decade for the second shell. The disorder parameters determined by the fits also show a slightly increasing trend with the exposure, however with a large scatter.

While it is well known from the literature that oxygen occupies octahedral interstitial positions in the body-centred-cubic lattice of Nb (see, for example, Kofstad, 1966 ▸; Blanter & Khachaturyan, 1978 ▸; Dosch *et al.*, 1986 ▸), the position of nitro­gen dissolved within the Nb host lattice is still under debate; however the octahedral interstitial site appears to be most likely (Richter *et al.*, 1976 ▸; Clenny & Rosa, 1980*b* ▸; Schubert *et al.*, 1984 ▸; Dosch *et al.*, 1984 ▸; Metzger *et al.*, 1985 ▸; Dangwal Pandey *et al.*, 2018 ▸). The measurements of the prepared samples at cryogenic temperatures (Fig. 11 ▸) are compatible with nitro­gen atoms occupying octahedral lattice sites. As the octahedral site is located directly between the second nearest neighbours in a distance *R*_2_, increasing amounts of nitro­gen on octahedral sites directly force *R*_2_ to increase. The nearest neighbour distance *R*_1_ is only indirectly affected, since there is no space to accommodate a nitro­gen atom between directly neighbouring Nb atoms, and thus, as a consequence, the increase of *R*_1_ with nitro­gen exposure is less pronounced as observed. Therefore, the results presented here indicate the feasibility for a direct determination of the crystallographic position of the adsorbed and dissolved N atoms. The data evaluation necessary for this is, however, beyond the topic of this work and will be discussed in a forthcoming paper (Rothweiler *et al.*, 2021 ▸).

## 5. Conclusions   

The structural changes of thin niobium foils during heat treatments at temperatures of 900°C were *in situ* investigated by X-ray absorption fine-structure measurements in a newly constructed high-temperature cell. The cell allows temperatures of up to 1200°C under clean high-vacuum conditions as well as controlled treatments employing well defined gas environments. Experiments performed under vacuum revealed that no structural changes occurred; in particular the Nb samples were not oxidized even after prolonged heating at 900°C for several hours. According to detailed fits of the EXAFS data obtained after re-cooling the samples to room temperature, the short-range-order structure parameters (*i.e.* the nearest distances, coordination numbers and disorder parameters) determined after those heat treatments resemble those of pristine Nb metal foils.

According to the quantitative analysis of time-resolved *in situ* XANES measurements by linear-combination fits, the exposure of the heated Nb foils to dilute air (pressures between 1 × 10^−5^ mbar and 1 × 10^−3^ mbar) revealed the formation of Nb(II) oxide (NbO). The determined oxidation rate dramatically increases with the applied air pressure, from values of about 1 nm min^−1^ for an air pressure of 10^−5^ mbar, to ∼200 nm min^−1^ for *p* = 10^−3^ mbar. Thus it can be anticipated that well defined gas exposures at high temperatures are possible with the new cell, since it offers a base pressure in the range of 10^−6^ mbar and an accordingly further reduced oxidation of niobium or other reactive materials, *i.e.* clean and well defined working conditions. A more quantitative analysis of the EXAFS data of the oxidized samples after cool-down to room temperature is part of future work, to foster the derived conclusions.

As a first case study, the Nb foils were exposed to nitro­gen atmospheres, and the performed *in situ* experiments clearly show that nitro­gen diffuses into the heated Nb, obeying a saturation level that corresponds well to equilibrium concentrations reported in the literature. Preliminary, ex situ EXAFS measurements at cryogenic temperatures allow more detailed insights into the structure of the nitro­gen-treated Nb foils: at cryogenic temperatures, lattice vibrations are suppressed to a larger extent, enhancing the EXAFS amplitudes χ(*k*)*k* ^3^ substantially and making the bond length determinations much more precise. We have observed a slight increase of the Nb—Nb bond lengths of the first two shells *R*_1_ and *R*_2_ during N_2_ exposure, and a slight increase of the disorder. The systematic variations observed for the N_2_ treated Nb are compatible with nitro­gen atoms on octahedral lattice sites. The quantitative modelling of the observed structural changes will be a topic of future investigations, in order to clearly identify the positions of N within the Nb host lattice. Furthermore, the influence of co-adsorbed oxygen, air as well as hydrogen on the nitro­gen uptake will also be studied.

## 6. Related literature   

The following references, not cited in the main body of the paper, have been cited in the supporting information: Basinski *et al.* (1955 ▸); Beckhoff *et al.* (2007 ▸); Berger *et al.* (2010 ▸); Olsson & Landolt (2003 ▸); Strauß *et al.* (2020 ▸); Von Polheim (2020 ▸); Von Polheim *et al.* (2021 ▸).

## Supplementary Material

Supporting figures S1 to S8. DOI: 10.1107/S1600577520013557/ok5025sup1.pdf
