# Supplementary material for: High-temperature treatments of niobium under high vacuum, dilute air- and nitro­gen-atmospheres as investigated by in situ X-ray absorption spectroscopy
Source: J Synchrotron Radiat. 2021 Jan 1;28(Pt 1):266–77. doi: 10.1107/S1600577520013557 (PMC7842222; doi:10.1107/S1600577520013557)
Supplement: Supplementary file 1 [file s-28-00266-sup1.pdf]

JOURNAL OF SYNCHROTRON RADIATION

Volume 28 (2021)

Supporting information for article:

High temperature treatments of niobium under high vacuum, dilute air- and nitrogen-atmospheres as investigated by in-situ X-ray absorption spectroscopy

Jonas Klaes, Patrick Rothweiler, Benjamin Bornmann, Ralph Wagner and Dirk Lützenkirchen-Hecht

Supplemental information for

"High temperature treatments of niobium under high vacuum, dilute air- and nitrogen-atmospheres as investigated by in-situ X-ray absorption spectroscopy" submitted to J. Synchroton Rad.

Authors

Jonas Klaes[a], Patrick Rothweiler[a], Benjamin Bornmann[a], Ralph Wagner[a] and Dirk Lützenkirchen-Hecht[a]*

[a]Fakultät 4 - Mathematik und Naturwissenschaften, Bergische Universität Wuppertal, Gauß-Str. 20, Wuppertal, 42097, Germany

Correspondence email: dirklh@uni-wuppertal.de

This supplement contains additional information for the above-mentioned manuscript. We report Nb K-edge XANES spectra of several Nb-O reference compounds as well as niobium nitride NbN and their edge positions (Figure S1).

The XANES spectrum of the Nb-sample heat-treated at 900 °C for one hour in the commercial cell was fitted with different linear combinations of Nb-metal, NbO and Nb_2O_5, and quantitative results of the determined compositions are reported in Figure S2. As can be seen, the XANES spectrum can be fitted by using any of the oxides with a reasonable fit quality, however the R-factor of the fit improves if NbO is employed (R=1.84 x 10^{-4}) instead of Nb_2O_5 with R=2.47 x 10^{-4}, and the fit quality gets even superior, if both oxides are used (R=1.11 x 10^{-4}). Thus the presence of both oxides is likely.

The quality of the k^3-weighted EXAFS fine structures $\chi(k)*k^3$ obtained in-situ at different temperatures is depicted in Figure S3. At room temperature, clear and almost noise-free oscillations are detected for k > 16 Å^{-1}, and even for k > 18 Å^{-1} the noise level is only moderate. For the data evaluations presented here, we have used the k-range from 2.2 – 14.5 Å^{-1} to ensure best possible data quality. As can be seen in Figure S3, the amplitude of the EXAFS $\chi(k)*k^3$ decreases substantially with increasing temperature in the range from 400 – 800 °C, and an increased noise level for larger k-values above ca. 10 Å^{-1}, and thus, we have used k^2-weighted data for the analysis of the EXAFS at elevated temperatures.

Figure S4 (a) shows grazing incidence X-ray measurements of a heat treatment of a polished stainless steel sample (1.4301 steel (AISI 304, X5CrNi18-10), i.e. Fe based with typically 18-20 wt. % Cr and

IMPORTANT: this document contains embedded data - to preserve data integrity, please ensure where possible that the IUCr Word tools (available from http://journals.iucr.org/services/docxtemplate/) are installed when editing this document.

8-11 wt.% Ni) under vacuum. Using X-ray reflectometry at a photon energy of 9000 eV with ionization chambers as detectors for incident and reflected X-ray intensities, a critical angle of about $\phi_c \approx 0.3°$ was found for the pristine steel sample, well in agreement with calculations. Above the critical angle, a small reflectivity maximum can be observed at about $\phi \approx 0.5°$, which can be explained by the presence of a native oxide layer of about 15-20 nm thickness on top of the steel surface. By employing a heat treatment in vacuum using temperatures of about 1000 °C, a de-oxidation of the steel surface is possible according to previous studies (Lützenkirchen-Hecht et al., 2016, Strauß et al., 2020). Accordingly, the reflectivity feature at about $\phi \approx 0.5°$ is absent after the heat treatment. During the reflectivity measurements we have simultaneously measured X-ray fluorescence (XRF) spectra for each data point, using a silicon drift detector (Amptek XR-100SDD, Bedford, USA) collecting the X-ray photons on the entrance window of the high-temperature cell. A typical example of an XRF spectrum obtained within 10 s of integration time for an incidence angle of $\phi = 0.2°$ is presented in the inset of Figure S4 (a). Clear signatures of the K_α- and K_β-peaks of Cr (5410 eV and 5947 eV), Fe (6404 eV and 7058 eV) and Ni (7478 eV and 8265 eV) are detected. Those signals were integrated, and by employing the photoionization cross sections (Berger et al., 2010) and the energy-dependent detector response function, an estimate for the quantitative composition was determined for each incidence angle, having in mind however that a much more sophisticated analysis of the XRF signals is required to obtain quantitative results (see e.g. Beckhoff et al., 2007). The angle-dependent results are compiled in Figure S4 (b). First of all it should be noted, that the determined bulk concentrations of Cr, Fe and Ni are well within the specification of the AISI 304 (X5CrNi18-10) steel, and thus the data analysis appears to be appropriate for the purpose here.

As can be seen in Figure S4 (b), the surface of the native steel sample is enriched in iron. Compared to the bulk concentration of about 74 wt.%, up to 77 wt.% were found for incidence angles of about 0.2° – 0.3°, where the penetration depth of the X-rays is in the order of some few nm only (Parratt, 1954). With values of 17-18 wt.%, the concentration of Cr is also slightly enhanced compared to the measured bulk value of 15 wt.%, however Cr seems to be more enriched in the outer surface of the passive layer on the steel (i.e. smaller incidence angles), while Fe seems to be enriched at the inner interface of the oxide layer to the bulk metal. Ni is substantially depleted in the oxide, with concentrations of about 7-8 wt.% compared to ca. 11 wt.% in the bulk. Those observations agree qualitatively with the structure of passive layers on steels obtained by surface analytical methods such as X-ray photoelectron spectroscopy (see, e.g. Olsson & Landolt, 2003). After the heat treatment in vacuum for 1 h at 900 °C, the concentration profiles substantially change, i.e. Fe is depleted from the surface of the sample with values as low as 69 wt.%, while Cr reaches concentrations of about 24 wt.%. The depletion of Ni in the surface seems to be slightly increased compared to the pristine steel, however with values still smaller than the bulk. These results thus clearly support the assumption that surface sensitive X-ray experiments can be in-situ performed in the high-temperature cell.

Figure S5 shows transmission mode XANES and XRD measurements on an iron foil performed in the vicinity of the Fe K-edge (7112 eV) within the high-temperature cell for different temperatures at beamline P64 under clean high-vacuum conditions. As can be seen, the foil reveals XANES features typical for the bcc-structure at room temperature and 876 °C. Due to the increased amplitude of the lattice vibrations and the lattice expansion, the XANES oscillations slightly decreased for T=876 °C. However, for T=923 °C, the XANES features have dramatically changed, due the bcc – fcc phase transition at T ≈ 915 °C (Basinski et al, 1955). The Quick-EXAFS monochromator was oscillating with 1 Hz in the range from ca. 6950 eV to 7800 eV for the measurement of the X-ray absorption fine structure data. The energies in the pre-edge range, i.e. in the time domain when the monochromator oscillation changes its direction, were used for the collection of the diffraction data presented in the insets in Figure S5. The diffractograms were thereby measured within ca. 150 ms for energies of ca. 7000 eV (corresponding to an X-ray wavelength of 1.77 Å), employing a Pilatus 100 K detector (Dectris, Baden, Switzerland) with an active area of 33.5 x 83.8 mm^2 and 197 x 487 pixels in a distance of ca. 250 mm from the sample. Bragg-angles around 50° in 2Θ were applied, so that the (110)-reflection of bcc Fe with Bragg angles of about 52° at room temperature and ca. 51.1° at 900 °C as well as the (111) fcc-reflection with ca. 49.8° can all be monitored on the 2D-detector. In the inset of Figure S5, a continuous Debye-Scherrer-ring related to the bcc (110)-reflection can be seen for the room-temperature measurement. However, at about 915 °C, i.e. close to the temperature of the bcc-fcc phase transition, both the (110) bcc reflection (shifted to smaller Bragg-angles by about 1°), as well as signatures of the (111) fcc structure at even smaller angles can be simultaneously observed. In agreement with the time-resolved XANES measurements, there exists a time and temperature range, in which both the bcc and the fcc structure can be detected, indicating that both phases are present in the sample simultaneously (von Polheim, 2020). Due to the presence of isolated diffraction spots instead of a continuous ring, it is obvious that recrystallization effects occurred during the heat treatment at elevated temperatures, which can be analyzed and interpreted consistently with the spectroscopic information obtained simultaneously by the XANES data. More details will be given in a forthcoming publication (von Polheim et al, 2020). Thus in-situ diffraction studies at high temperatures using clean high vacuum conditions as well as well-controlled gaseous atmospheres are feasible, e.g. for investigations of grain growth and grain reorientation, phase transitions and their kinetics.

Figure S6 shows linear combination fits of Nb K-edge XANES spectra obtained from a 8 μm Nb foil after heating at 900 °C under vacuum for different process times as indicated. The measurements were performed after cooling to room temperature, and the LC-XANES fitting suggests only a very slight oxidation of the Nb foils. From the obtained oxide fractions, an oxide growth rate of ca. 35 ± 10 nm/h results, i.e. a negligible oxidation for typical process times in the range of an hour.

In Figure S7, a two shell model was applied to the Fourier-transformed EXAFS data obtained after a one hour heat-treatment for 900 °C in the Anton Paar cell (compare Figure 1 of the main manuscript), neglecting the effects of oxidation in the fit model. The reduced amplitude corresponds to a substantially decreased value for S_0^2 = 0.79 ± 0.03, which is substantially lower compared to the average value obtained after a heat treatment in the new cell (S_0^2 = 0.93 ± 0.03), even if the confidence interval is taken into account (compare to Figure 5 of the main manuscript). Furthermore, a substantial increase of the inner potential shift derived from the fit was noticed (i.e. ΔE_0 = 4.1 ± 1.1 eV for the sample treated within the Paar cell, compared to ΔE_0 = 3.4 ± 0.8 eV in the new cell), which may also hint to a slight oxidation of the Nb foil during the heat treatment in the Paar cell.

Finally, different linear combination XANES fits of Nb-foils treated in air are shown in Figure S8. For comparison with the fit using NbO reference data shown in the main manuscript (Figure 7), here NbO_2 and Nb_2O_5 references were used together with Nb metal, leading to an overall substantially worse fit of the data and larger deviations between the experimental data and the fits. The fit residuals amount to R = 3.5 x 10^{-3} for the fit using Nb-metal and Nb_2O_5, R = 2.6 x 10^{-3} for the fit using Nb-metal and NbO_2, in comparison to R = 4.0 x 10^{-4} for the fit using Nb-metal and NbO.

Supplemental Figures and Figure captions:

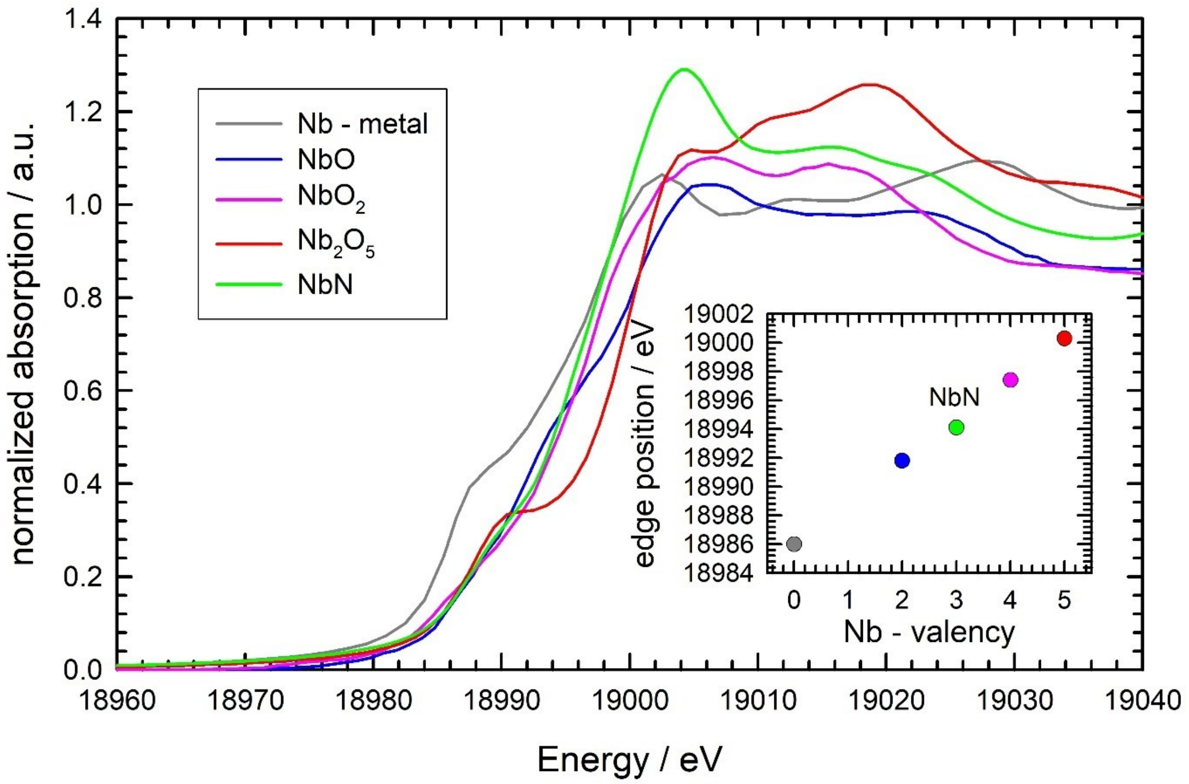

Figure S1 Normalized Nb K-edge XANES spectra of several Nb reference compounds as indicated. In the inset, the edge position determined by the first inflection point of the spectra is presented as a function of the formal chemical valency of the X-ray absorbing Nb-atoms. The colour code in the inset belongs to the spectra.

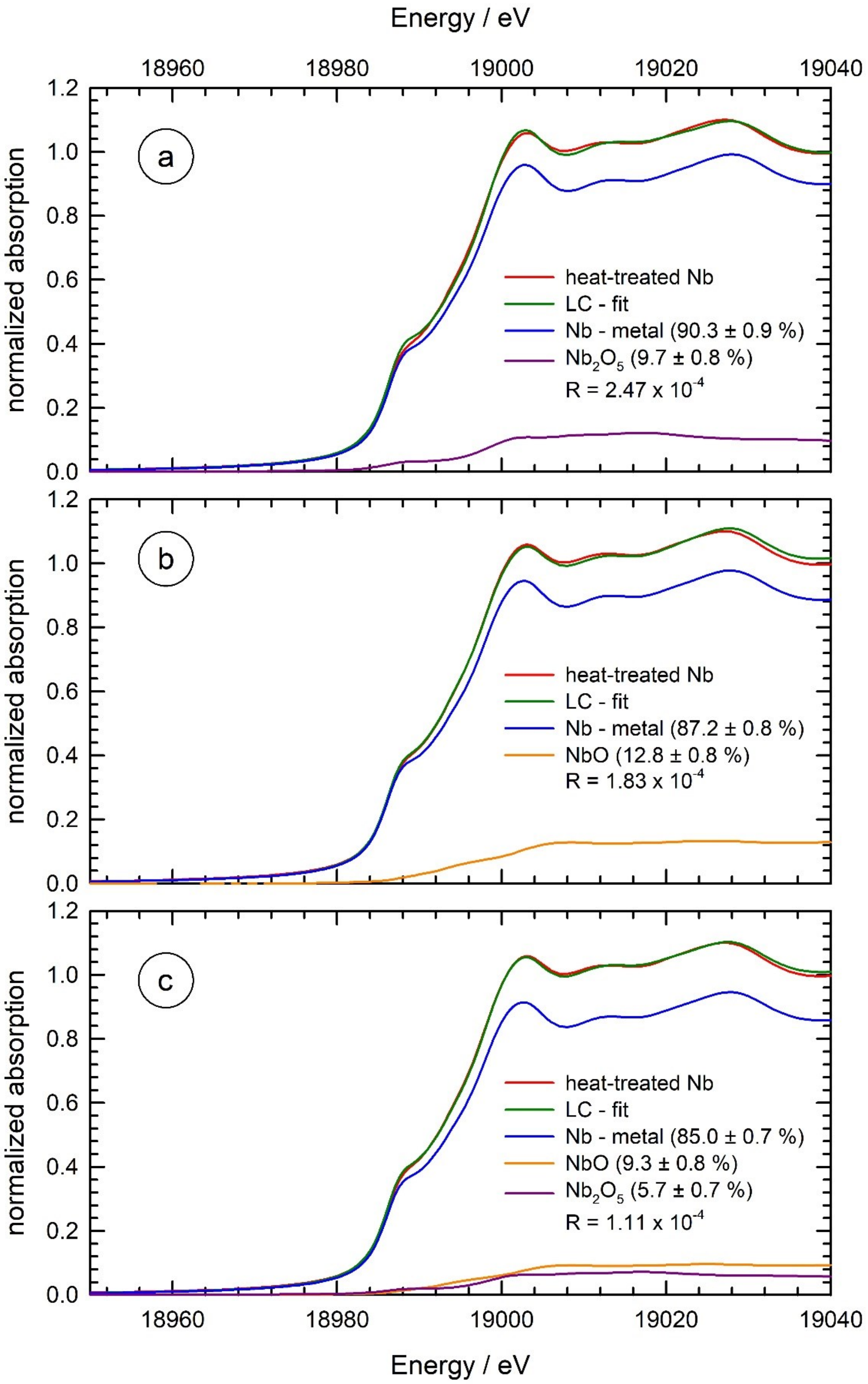

Figure S2 Linear-combination fits of the XANES spectrum of a Nb foil after a heat treatment in an Anton Paar DHS 1100 cell at 900°C for one hour, and cooling-back to room temperature using (a) Nb metal and Nb_2O_5, (b) Nb metal and NbO, and (c) Nb metal, NbO and Nb_2O_5 for the modelling of the experimental XANES data. The composition and the R-factors of the fits are given in each figure. The fits were performed between 18960 eV and 19040 eV.

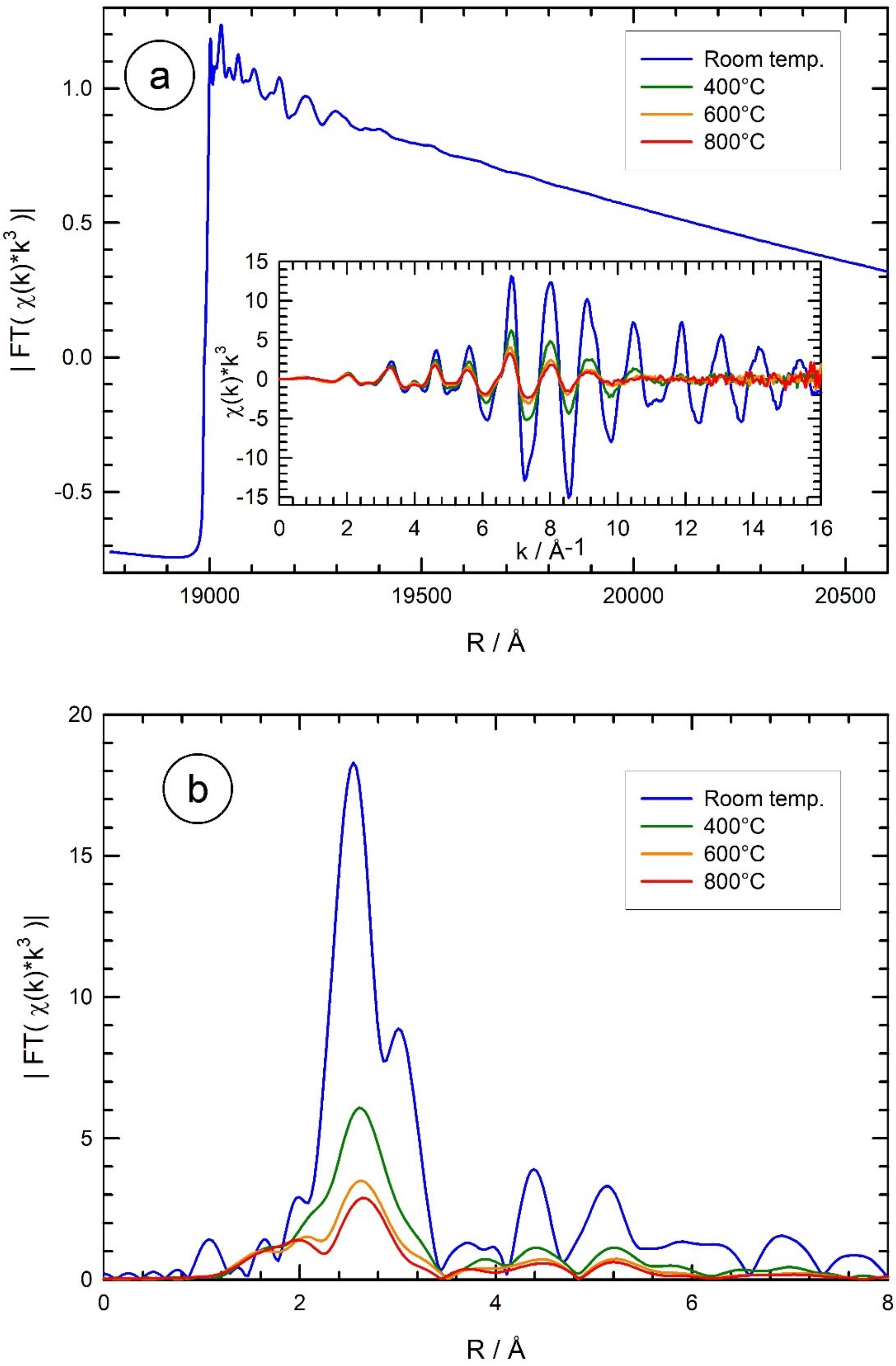

Figure S3 Examples of in-situ transmission mode EXAFS experiments of a Nb foil in vacuum at elevated temperatures. (a) Raw absorption data measured at room temperature within ca. 30 minutes of acquisition at DELTA BL10 for a scan from 18750 eV to 20600 eV. As can be seen in the inset, almost noise-free fine structure data $\chi(k)*k^3$ were recorded up to 16 $Å^{-1}$ and more at room temperature, while the amplitude of the EXAFS oscillations strongly decrease with increasing temperature, so that for T = 800 °C almost all oscillations are absent for $k > 12$ $Å^{-1}$, and a substantial increase of noise on the data. (b) Magnitude of the Fourier-transform of the k^3-weighted fine structure data $\chi(k)*k^3$ for different temperatures as indicated (k-range for the FT: $2.0 < k < 14.5$ $Å^{-1}$).

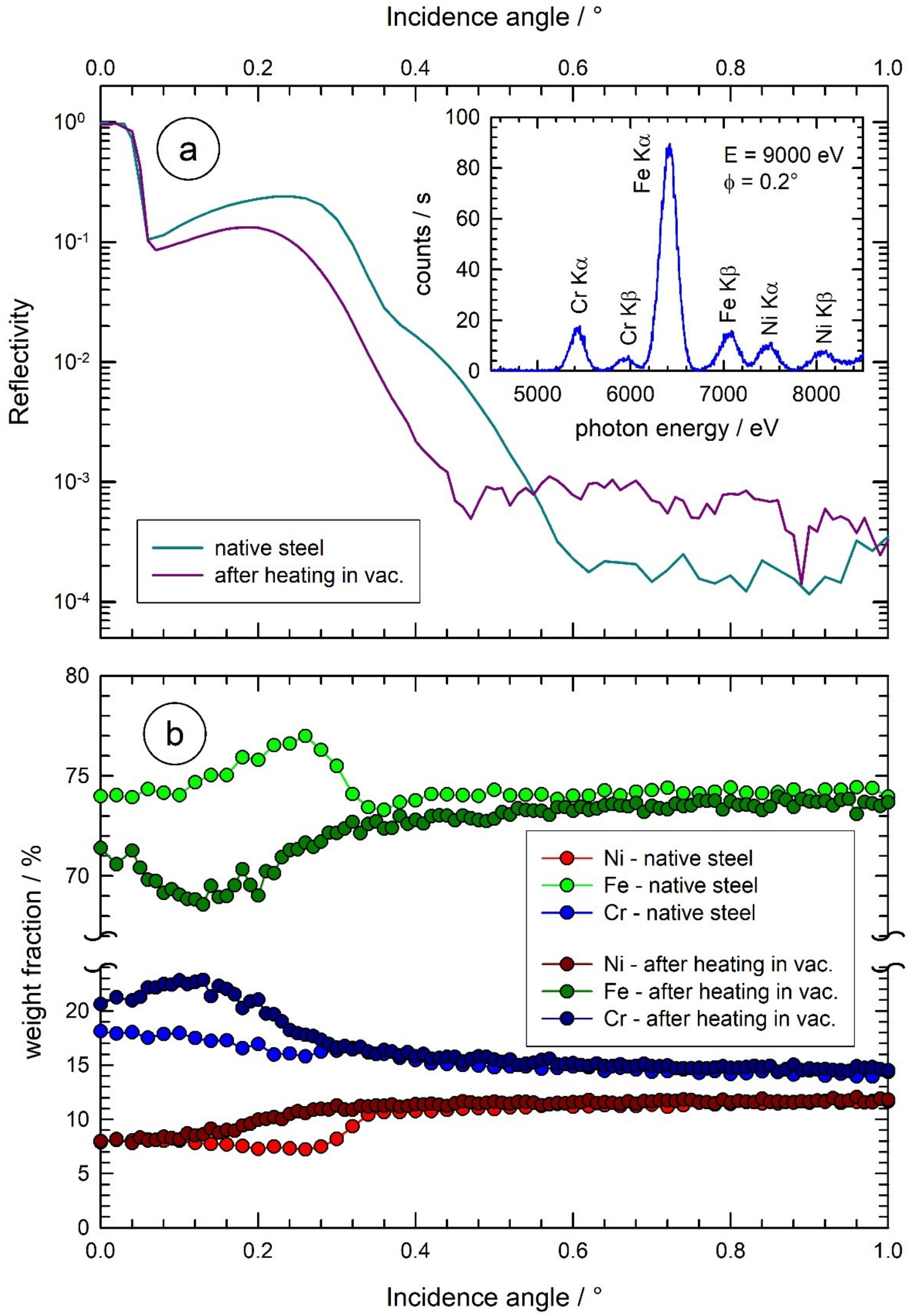

Figure S4 Grazing incidence X-ray study (excitation energy E=9000 eV) of a heat treatment of 1.4301 steel (AISI 304, X5CrNi18-10) in vacuum. (a) Angular dependence of the X-ray reflectivity as measured with an ionization chamber. In the inset, the X-ray fluorescence of the pristine steel sample excited for an incidence angle $\phi = 0.2°$ with contributions of Cr, Fe and Ni is depicted. The angular dependence of the fluorescence yield is measured as a function of the incidence angle ϕ. (b) Weight fractions of the metals calculated from the fluorescence data using the photoionization cross sections before and after a heat treatment in vacuum for 1 h at 900 °C. The decrease of the Fe contributions and the increase of the Cr concentration in the near surface region (small incidence angles $\phi \leq 0.25°$) can clearly be seen.

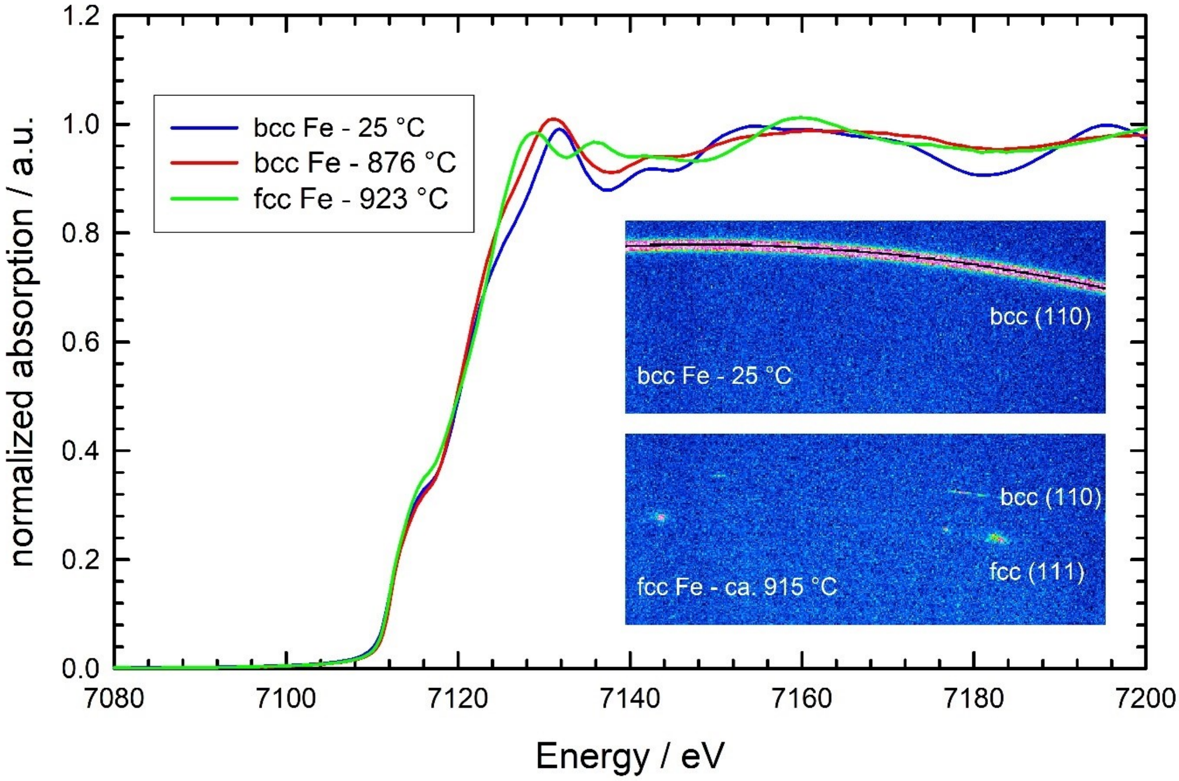

Figure S5 In-situ, transmission mode XANES spectra obtained from a Fe metal foil at various temperatures as indicated. Below 915 °C, Fe is in a bcc structure, while above the fcc phase is stable. In the insets, X-ray diffraction patterns (ca. 7000 eV photon energy) measured simultaneously on a Pilatus 100 K detector in a distance of ca. 250 mm to the Fe-foil within the high-temperature cell are presented for two temperatures. At 915 °C, diffracted intensities characteristic for both the fcc and the bcc structure are detectable.

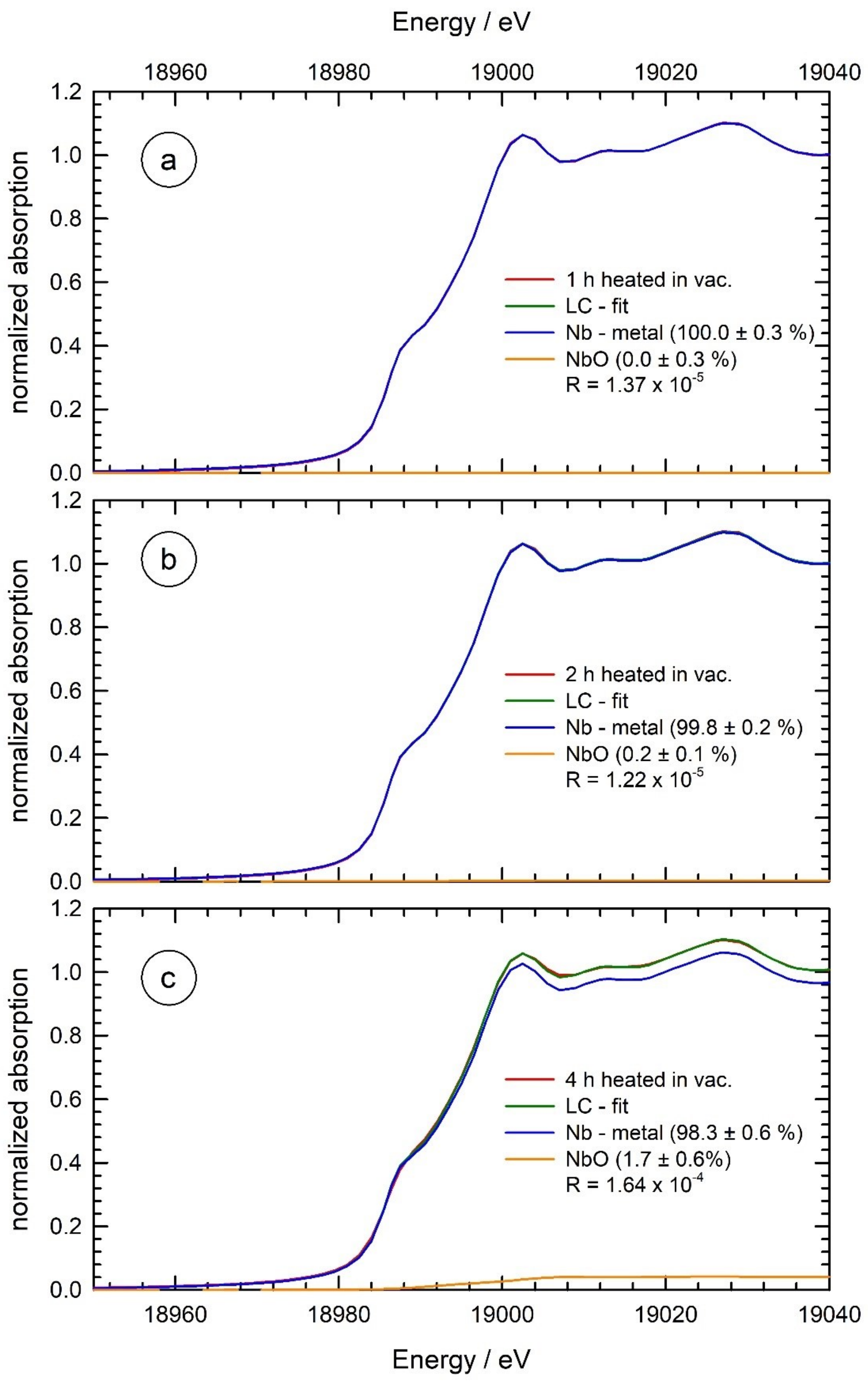

Figure S6 Linear-combination fits of the XANES spectrum of a Nb foil after a heat treatment under vacuum in the new process chamber at 900°C for (a) one hour, (b) two hours and (c) four hours, and cooling-back to room temperature using Nb metal and NbO. The determined compositions and the R-factors of the fits are given in each figure. The fits were performed between 18960 eV and 19040 eV.

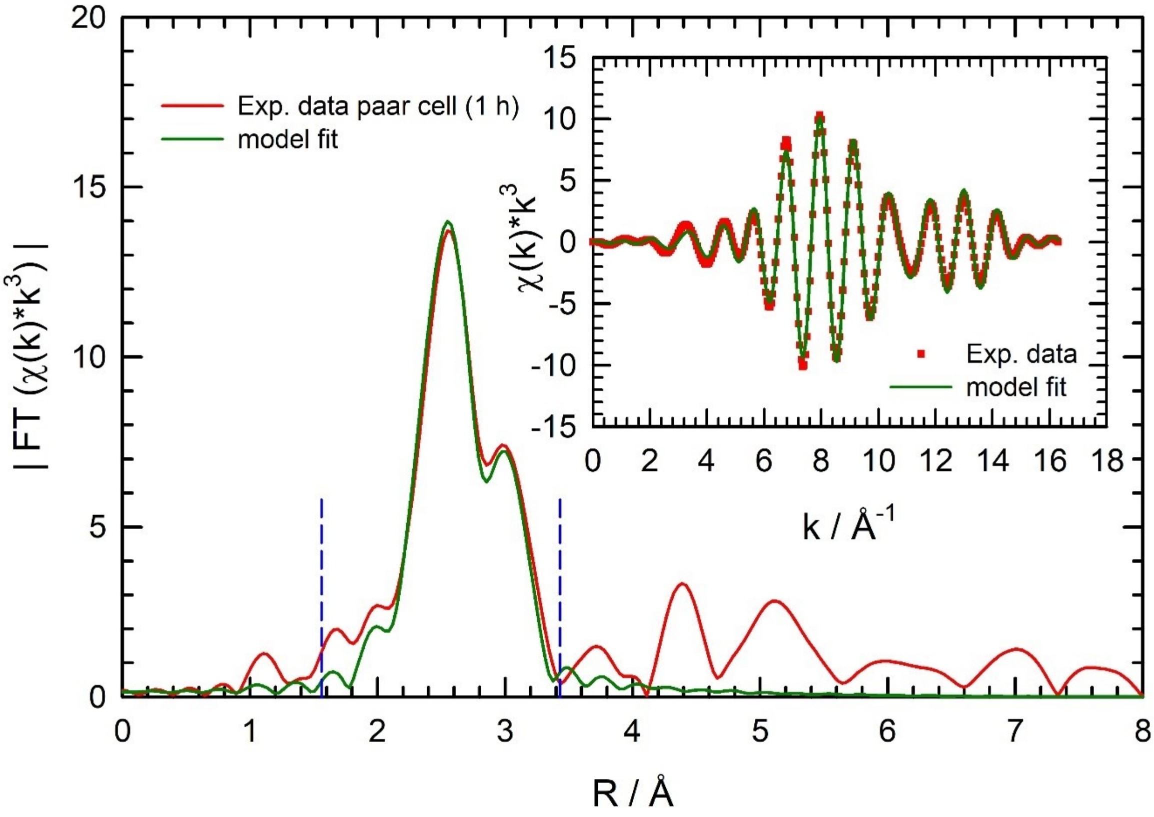

Figure S7 Fit of the EXAFS data obtained from a 25 µm thick Nb foil after an one hour heat treatment at 900 °C under vacuum by employing a two-shell model using the first and second Nb-Nb coordinations of the bcc-lattice; the experimental data (—) as well as the fit (—) are shown. The data from 1.5 Å to 3.4 Å in the FT (as indicated by vertical dotted blue lines) were back-transformed into k-space, and are displayed in the inset for the experimental data (■) and the fit (—).

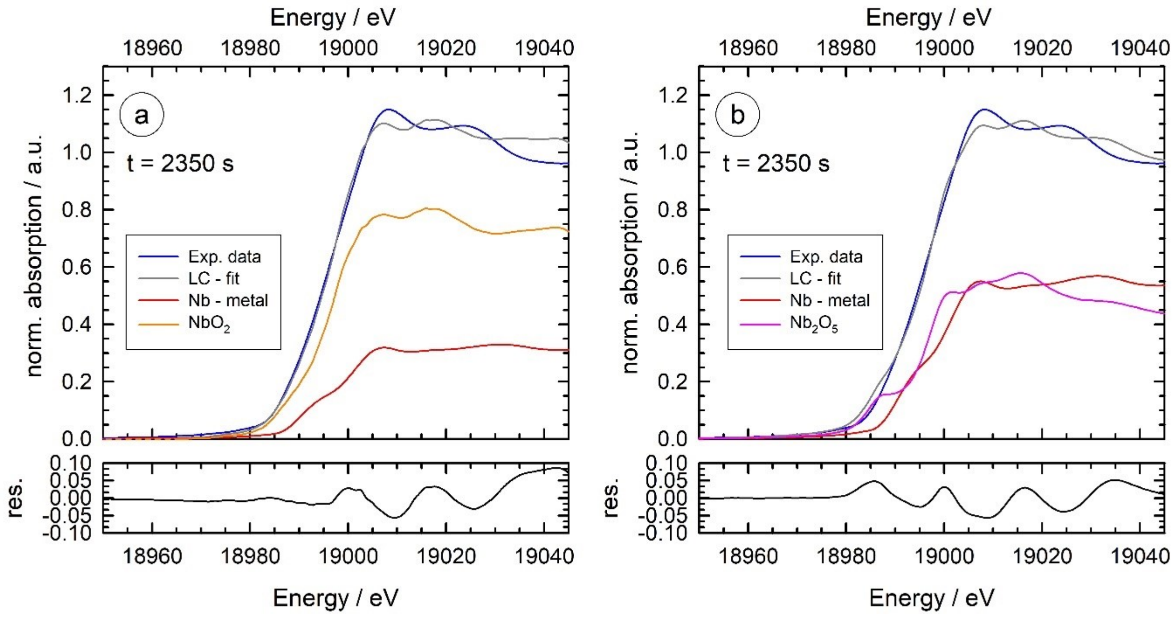

Figure S8 Examples of linear-combination XANES fits for a sample oxidized in an air atmosphere of 10^{-3} mbar for 2350 s. In (a) XANES spectra of Nb-metal and NbO_2 were used for the modelling, while in (b) Nb-metal and Nb_2O_5 were employed. The fits were performed between 18960 eV and 19045 eV. In the lower panels, the difference between the experimental data and the fit are presented. Note that the fit errors are substantially larger compared to the fits using NbO as reference (Figure 7 of the main manuscript).

References

Basinski, Z.S., Hume-Rothery, W. & Sutton, A.L. (1955). *Proc. Royal. Soc. A*, **229**, 459-467. https://doi.org/10.1098/rspa.1955.0102.

Beckhoff, B., Fliegauf, R., Kolbe, M., Müller, M., Weser, J. & Ulm, G. (2007). *Anal. Chem.*, **79**, 7873-7882. https://doi.org/10.1021/ac071236p.

Berger, M.J., Hubbell, J.H., Seltzer, S.M., Chang, J., Coursey, J.S., Sukumar, R., Zucker, D.S. & Olsen, K. (2010). NIST Standard Reference Database 8 (XGAM). https://dx.doi.org/10.18434/T48G6X.

Lützenkirchen-Hecht, D., Wulff, D., Wagner, R., Holländer, U., Maier, H.J. & Frahm, R. (2016). *J. Phys.: Conf. Ser.*, **712**, 012047 (1-4). https://doi.org/10.1088/1742-6596/712/1/012047.

Olsson, C.-O.A. & Landolt, D. (2003). *Electrochim. Acta*, **48**, 1093-1104. https://doi.org/10.1016/S0013-4686(02)00841-1.

Parratt, L.G. (1954). *Phys. Rev.*, **95**, 359-369. https://doi.org/10.1103/PhysRev.95.359.

Strauß, C., Wegewitz, L., Schöler, S., Holländer, U., Möhwald, K. & Maus-Friedrichs, W. (2020). *Steel Res. Int.*, **91**, 1900568. https://doi.org/10.1002/srin.201900568.

Von Polheim, S. (2020). MSc. thesis (in German), Wuppertal University, Wuppertal, Germany.

Von Polheim, S., Bornmann, B., Frahm, R., Eckelt, F. & Lützenkirchen-Hecht, D. (2020). In preparation.
